# MRI-based radiomics for noninvasive prediction of T790M resistance mutation in lung cancer spinal metastases: an exploratory study

**DOI:** 10.3389/fcell.2025.1673498

**Published:** 2025-10-29

**Authors:** Xiaoya Zhou, Jiashi Zheng, Hua Ai, Chunna Yang, Jiahui Wang, Yiyao Sun, Lijun Jin

**Affiliations:** ^1^ Jining Key Laboratory of Pharmacology, School of Basic Medicine, Jining Medical University, Jining, Shandong, China; ^2^ The First Clinical College, China Medical University, Shenyang, Liaoning, China; ^3^ School of Intelligent Medicine, China Medical University, Shenyang, Liaoning, China

**Keywords:** bone metastases, radiomics, intratumoral heterogeneity, targeted therapy, interpretability

## Abstract

**Background:**

The T790M mutation is a significant mechanism of acquired resistance to EGFR-TKIs in non-small cell lung cancer (NSCLC). Its noninvasive detection in spinal metastases remains challenging due to tumour heterogeneity and limitations of current diagnostic methods. This study aimed to develop an MRI-based radiomics model derived from spinal metastases to non-invasively predict T790M resistance mutations in NSCLC patients, by incorporating intratumoral spatial heterogeneity.

**Methods:**

One hundred ten EGFR-mutant NSCLC patients with spinal metastases (80 from Center 1, 30 from Center 2) underwent T1W and T2FS MRI scans. Spinal lesions were partitioned into phenotypically consistent subregions using patient- and population-level clustering based on local entropy to capture spatial heterogeneity. Radiomic features were extracted from each subregion, and reproducibility was assessed using the intraclass correlation coefficient (ICC >0.80). Significant features were selected via the Mann–Whitney U test and LASSO regression, and logistic regression models were constructed for each subregion and MRI sequence. A multi-sequence regional fusion model was subsequently developed based on the best-performing subregion. Model performance was evaluated by AUC, sensitivity, and specificity in both internal and external validation cohorts. SHAP analysis was conducted to interpret feature contributions.

**Results:**

Models based on inner subregions with higher heterogeneity outperformed those from marginal or whole-tumor regions. The fusion model combining T1W and T2FS features achieved AUCs of 0.916 (training), 0.867 (internal validation), and 0.839 (external validation). SHAP analysis identified key textural features associated with the T790M mutation.

**Conclusion:**

Subregion-based MRI radiomics enables accurate, noninvasive prediction of T790M mutations in NSCLC spinal metastases. This subregion-based MRI radiomics model, to our knowledge, is the first to non-invasively predict T790M resistance mutations in spinal metastases by integrating spatial heterogeneity and SHAP interpretability. This subregion-based MRI radiomics model is exploratory and showed a consistent trend toward improved discrimination and net benefit.

## 1 Introduction

Lung cancer remains the leading cause of cancer-related mortality worldwide, with non-small cell lung cancer (NSCLC) accounting for approximately 85% of cases (Thai et al., 2021; Sung et al., 2021). Among NSCLC patients, spinal metastases are a frequent complication, which significantly deteriorates prognosis and quality of life (Shi et al., 2021). Bone metastasis interferes with normal bone remodeling processes, resulting in serious complications including refractory bone pain, pathological fractures, hypercalcemia, and spinal cord compression, all of which substantially compromise the quality of life in affected patients (Coleman, 2001).

Epidermal growth factor receptor (EGFR) tyrosine kinase inhibitors (TKIs) have revolutionized the treatment of EGFR-mutant NSCLC, substantially improving clinical outcomes (Shirley and Keam, 2022; Wang et al., 2022). However, acquired resistance, predominantly driven by the T790M mutation, a secondary point mutation occurring at amino acid 790 of the EGFR gene (Pao et al., 2005), emerges in over half of resistant cases, posing a major therapeutic challenge. In the early and middle stages, patients generally exhibit a favorable response to targeted therapy. However, the majority of them develop resistance after 8–13 months of treatment with first- or second-generation tyrosine kinase inhibitors (TKIs), such as gefitinib and erlotinib (Mok et al., 2017). The T790M mutation often necessitates the use of third-generation TKIs, such as Osimertinib, which are specifically designed to overcome T790M-mediated resistance (Cross et al., 2014; Ramalingam et al., 2020). Consequently, early and accurate detection of the T790M mutation status is critical for optimizing treatment strategies and improving patient outcomes.

Currently, the clinical detection of T790M mutations primarily relies on tumor tissue biopsy or testing for circulating tumor DNA (ctDNA) (Rolfo et al., 2021). These methods are used to determine the mutation status to a certain extent and guide the use of third-generation TKI drugs (Soria et al., 2018). However, tissue biopsy is an invasive procedure with sampling bias and the risk of complications, while ctDNA detection is limited by insufficient sensitivity, technical complexity, and high cost (Siravegna et al., 2017). Moreover, intratumoral heterogeneity makes it difficult for these methods to comprehensively capture the mutational landscape of bone metastases (Dagogo-Jack and Shaw, 2018; Kobayashi and Tan, 2023; Lim and Ma, 2019). Although the use of imaging for genetic assessment is increasing, noninvasive techniques such as MRI can only provide morphological and functional information (Lambin et al., 2017). Traditional imaging evaluation mainly relies on visible morphological features, lacking specific markers for assessing T790M drug-resistant mutations (Zhang et al., 2024b). The internal composition of bone metastases is highly complex, comprising tumor cells, residual bone tissue, and normal bone tissue, which results in significant differences within the internal regions of bone metastases (Clézardin et al., 2021; Coleman, 2001).

The emergence of radiomics methods has provided new ideas for quantitative image analysis (Lambin et al., 2017). By extracting high-dimensional features, radiomics can quantify intratumoral heterogeneity and reveal potential molecular characteristics (Gillies et al., 2016; Ibrahim et al., 2021). Recent studies have demonstrated that radiomics methods based on primary lung cancer imaging can effectively predict T790M resistance mutations following targeted therapy (Lu et al., 2024; Li et al., 2023; Zhang et al., 2024b). Fan et al. (2023a), Fan et al. (2023b) and Lv et al. (2023) have also developed MRI-based radiomics models using brain metastases from lung cancer to predict T790M mutation status.

These studies indicate that a variety of radiomic features are highly correlated with the T790M status in both lung cancer and distant metastases, which can be effectively mined through radiomics methods. Additionally, tumor heterogeneity—which encompasses the diversity of genetic, phenotypic, and microenvironmental characteristics—has attracted increasing attention due to its significant impact on diagnosis, treatment response, and clinical prognosis (Dagogo-Jack and Shaw, 2018; McGranahan and Swanton, 2017; Vitale et al., 2021). However, whether the spatial heterogeneity of lung cancer bone metastases is associated with acquired drug resistance after targeted therapy remains unclear, owing to a lack of relevant studies (Clézardin et al., 2021; Wu et al., 2021). Therefore, this study utilized a subregion-based radiomics analysis to investigate the relationship between intratumoral heterogeneity and T790M mutation status, aiming to provide technical support for elucidating resistance mechanisms and guiding personalized treatment decisions.

## 2 Materials and methods

### 2.1 Patients

**FIGURE 1:**
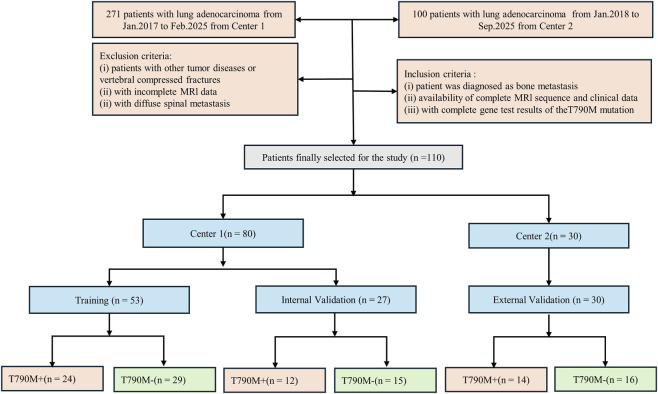
Flowchart of patient inclusion and exclusion criteria for the study, showing the selection process for NSCLC patients with spinal bone metastases based on eligibility and MRI data availability.

This retrospective study was approved by the Institutional Ethics Committee of Liaoning Cancer Hospital (Approval No. 20220806YG), with a waiver of informed consent. A total of 271 patients from Liaoning Cancer Hospital (Center 1), who were treated between January 2017 and February 2025, and 100 patients from Shengjing Hospital (Center 2), who were treated between January 2018 and September 2025, were initially enrolled. The T790M mutation status was identified in plasma ctDNA extracted from blood samples following treatment with first- or second-generation TKIs, based on pathological biopsy specimens of the primary tumor prior to treatment. Inclusion criteria were: (1) pathological diagnosis of non-small cell lung cancer (NSCLC) with imaging confirmation of spinal bone metastasis; (2) complete baseline MRI data, including T1-weighted imaging (T1W) and T2-weighted fat-suppressed imaging (T2FS); and (3) confirmed T790M mutation status based on tissue biopsy or ctDNA testing results. Exclusion criteria were: (1) presence of other malignant tumors, (2) incomplete or poor-quality MRI data, and (3) absence of T790M mutation gene testing results. Patients from Center one were divided into a training and internal validation group at a 2:1 ratio (stratified by T790M status), while patients from Center two were used as an independent validation group. Clinical characteristics were collected from hospital medical records. The detailed process of patient inclusion and grouping is shown in Figure 1. Clinical factors, including age, gender, smoking, carcinoembryonic antigen (CEA), performance status (PS) score, cytokeratin (CYFRA), and neuron-specific enolase (NSE), were obtained from the medical records.

### 2.2 MRI scanning methods and parameters

All MRI examinations at both centers were performed using a Siemens 3.0 T MRI scanner (Siemens Magnetom Trio, Erlangen, Germany). T1W and T2FS sequences were obtained. In center 1, the scanning parameters for the T1W sequence were as follows: repetition time (TR) = 500 m, echo time (TE) = 9 m, slice thickness = 4 mm, and inter-slice gap = 4.4 mm. For the T2FS sequence, TR = 3,000 m, TE = 78 m, slice thickness = 4 mm, and inter-slice gap = 4.4 mm. In center 2, the scanning parameters for the T1W sequence were as follows: repetition time (TR) = 514 m, echo time (TE) = 11 m, slice thickness = 4 mm, and inter-slice gap = 4.4 mm. For the T2FS sequence, TR = 3,000 m, TE = 87 m, slice thickness = 4 mm, and inter-slice gap = 4.4 mm.

Regions of interest (ROIs) were manually delineated on the spinal metastatic lesions by two radiologists with 15 and 10 years of experience, respectively, who were blinded to patients’ pathological results. The first radiologist performed the initial segmentation using ITK-SNAP software (version 3.6; www.itksnap.org), and the second radiologist independently reviewed and confirmed the segmentation.

### 2.3 Intratumor partitioning methods

The intratumoral partitioning method consisted of three steps. First, within each ROI, the MRI local entropy was calculated using small neighborhoods in the ROI (9 × 9 pixels, stride = 1 pixel), while also preserving the original pixel intensity; both entropy and intensity values were normalized (Z-score) within each ROI. Next, the ROI was divided into different subregions by the K-means (Gutman et al., 2013) clustering algorithm, mapping each pixel in the ROI into the two-dimensional feature space intensity, local entropy, and distance measured by Euclidean distance. Second, through K-means clustering, each ROI was clustered into 30 superpixels, with each superpixel’s intensity determined by the average intensity of all pixels within it. The number 30 was selected after comparing candidate values (20, 30, 40, 60) in early experiments evaluating within-cluster variance and inter-patient/inter-sequence consistency. Third, the superpixels of all patients were gathered together, and the similarity between and within patients was explored through hierarchical clustering using Ward linkage to realize group-level clustering. Using the k-means clustering algorithm, each tumor was divided into different subregions in space. In order to avoid the occurrence of a local optimal solution, the number of subregions (clusters K) is set from 2 to 10 (Pham et al., 2005). The optimal number of subregions is confirmed by using the Calinski-Harabasz (CH) index and Silhouette coefficient (Caliński and Harabasz, 1974).

### 2.4 Radiomics feature extraction and selection

The “pyradiomics” package was implemented on Python v.3.10 to extract radiomics features. Detailed information about the pyradiomics documentation and radiomics features can be found on the official website: https://pyradiomics.readthedocs.io/en/latest/index.html. A total of 1,967 radiomic features, including first-order, shape-based, texture (e.g., GLCM, GLRLM, GLSZM, NGTDM, and GLDM), and filtered image features, were extracted separately from each spinal metastasis subregion and the whole tumor region across two MRI sequences. A total of eight image filters, including wavelet, square, square root, local binary pattern (2D), Laplacian of Gaussian, logarithm, exponential, and gradient, were applied to the MR images. The resulting filtered images were subsequently used to extract first-order statistical and texture features. Before feature extraction, MR images were pre-processed including normalization, resampling, discretization, and filtering, detailed preprocessing protocols are provided in Supplementary Methods 1.

ComBat harmonization has been widely used to eliminate the effects of different scanners and protocols and to facilitate multicenter radiomics analysis. We used the neuroHarmonize implementation in Python v3.10 (https://github.com/rpomponio/neuroHarmonize/tree/master) to harmonize our extracted radiomics features per scanner without changing the feature definitions.

To ensure the reliability and reproducibility of the extracted features, intraclass correlation coefficients (ICCs) were calculated based on repeated feature extractions from a randomly selected subset of 30 patients. Features with ICC values greater than 0.80 were considered highly reliable and were retained for further analysis.

Subsequently, feature selection was conducted strictly within the training set and confined to the inner loop of a nested five-fold cross-validation framework to avoid data leakage (Demircioğlu, 2021). The Mann-Whitney U test was applied to the extracted feature using the “stats” package in R language Version 3.6. Features that have *P* < 0.05 were considered predictive and retained. Next, the least absolute shrinkage and selection operator (LASSO) logistic regression was used with the “glmnet” package in R to exclude irrelevant and redundant features.

### 2.5 Radiomics model construction and validation

Radiomics signatures (RSs) were constructed from weighted linear combinations of the selected features, which can be used to calculate a personalized score for each patient. The receiver operating characteristic (ROC) curve for the developed models was plotted with the scikit-learn v1.0 package in Python v3.10. The best cutoff value was determined by the maximum Youden index (Ruopp et al., 2008). The DeLong test was used to compare AUC differences between models, evaluating their predictive performance for T790M mutations. To evaluate clinical utility beyond discrimination metrics, we employed Decision Curve Analysis (DCA) (Vickers and Elkin, 2006) and calibration curves (Van Calster et al., 2019) to assess the net benefit across a range of threshold probabilities, comparing our model to default strategies such as “treat all” and “treat none.” Decision curve analysis is a widely accepted method for quantifying clinical value and can reveal benefits not captured by AUC alone.

### 2.6 Statistical analysis

**FIGURE 2:**
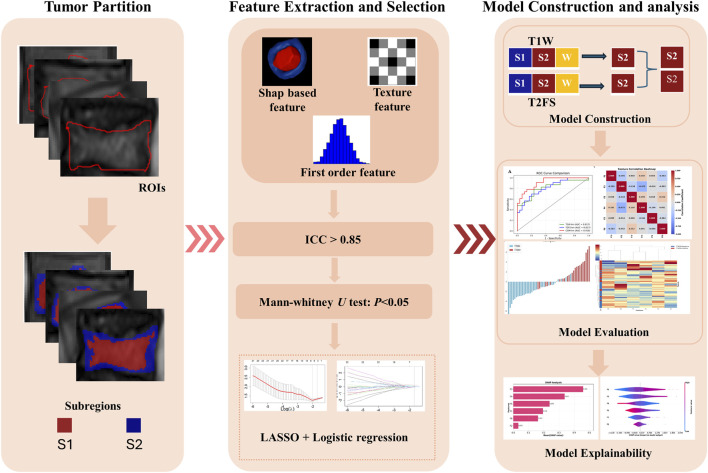
Workflow of the study, including tumor partition, radiomics feature extraction and selection, and model evaluation.

Statistical analysis included t-tests and Mann-Whitney U tests, depending on data distribution, to assess the differences in radiomics features between T790M-positive (T790M+) and T790M-negative (T790M-) patients. Clinical parameters were analyzed using Mann-Whitney U tests and chi-square tests. ROC analysis, using AUC as the primary metric, calculated the accuracy, sensitivity, and specificity to evaluate model performance across various data subsets. The optimal model for predicting T790M mutations was identified based on AUC comparison using the DeLong test. To further address potential class imbalance, we also reported area under the precision–recall curve (AUPRC), which focuses more on the minority (positive) class performance and may offer more actionable insight than AUC in the presence of imbalance (Brabec et al., 2020). Figure 2 illustrates the overall experimental workflow.

### 2.7 SHAP analysis

To interpret the construct RS, this study employed the “shap” package in Python for Shapley Additive exPlanations (SHAP). SHAP values were computed to assess the contribution of each selected feature to the T790M mutation predictions. Additionally, SHAP bar charts and beeswarm plots were generated to visualize feature importance and the direction of their impact, thereby offering an easy way to understand the model’s decision-making process.

### 2.8 Hardware and software resources

All computations were performed on a workstation equipped with an Intel® Core™ i7-8700K CPU, an NVIDIA GeForce RTX 1060 GPU (6 GB VRAM), and 16 GB system RAM. The operating environment included Python 3.10 and R 3.6, with key packages such as pyradiomics, scikit-learn (v1.0), glmnet, stats, shap, and neuroHarmonize.

## 3 Results

### 3.1 Patients’ characteristics

Table 1 summarizes the clinical and demographic characteristics of the enrolled patients, comparing those with and without the T790M mutation across various factors. In the training set, there were significant differences between the T790M+ and T790M-groups in terms of smoking status (*P* = 0.009). However, no significant difference was observed in age, gender, PS score, CEA, CYFRA, or NSE (all *P* > 0.05).

**TABLE 1:** Patients and their clinical characteristics for predicting T790M mutation.

Characteristic	Training set (n = 53)		*P*	Internal validation set (n = 27)		*P*	External validation set (n = 30)		*P*
	T790M+ (n = 24)	T790M– (n = 29)		T790M+ (n = 12)	T790M-(n = 15)		T790M+ (n = 14)	T790M-(n = 16)	
Age (Mean ± SD)	58.54 ± 7.90	60.55 ± 11.58	0.474	58.67 ± 9.08	60.27 ± 7.73	0.625	63.79 ± 6.67	62.06 ± 10.69	0.607
Gender			0.376			0.322			0.215
Male	7 (29.2%)	13 (44.8%)		4 (33.3%)	9 (60.0%)		3 (21.4%)	8 (50.0%)	
Female	17 (70.8%)	16 (55.2%)		8 (66.7%)	6 (40.0%)		11 (78.6%)	8 (50.0%)	
Smoking status			0.009*			0.589			0.783
Yes	11 (45.8%)	3 (10.3%)		2 (16.7%)	5 (33.3%)		2 (14.3%)	4 (25.0%)	
No	13 (54.2%)	26 (89.7%)		10 (83.3%)	10 (66.7%)		12 (85.7%)	12 (75.0%)	
PS score			0.689			0.196			0.161
0	4 (16.7%)	5 (17.2%)		0 (0.0%)	1 (6.7%)		2 (14.3%)	1 (6.2%)	
1	19 (79.2%)	21 (72.4%)		10 (83.3%)	13 (86.7%)		8 (57.1%)	14 (87.5%)	
2	1 (4.2%)	3 (10.3%)		2 (16.7%)	0 (0.0%)		4 (28.6%)	1 (6.2%)	
3	0 (0.0%)	0 (0.0%)		1 (6.7%)	1 (6.7%)		0 (0.0%)	0 (0.0%)	
CEA (mean ± SD)	136.60 ± 220.05	191.00 ± 312.02	0.476	61.05 ± 87.21	80.99 ± 114.23	0.622	73.18 ± 123.87	104.05 ± 209.82	0.634
CYFRA (mean ± SD)	8.55 ± 12.13	7.75 ± 8.55	0.781	6.21 ± 5.95	10.46 ± 13.21	0.313	25.96 ± 70.27	30.33 ± 100.09	0.892
NSE (mean ± SD)	32.20 ± 40.62	18.07 ± 8.96	0.074	21.04 ± 7.77	17.88 ± 10.75	0.402	24.28 ± 24.42	32.35 ± 65.74	0.668

*P < 0.05.

SD, standard deviation; PS, performance status; CEA, carcinoembryonic antigen; CYFRA, cytokeratin; NSE, neuron-specific enolase.

### 3.2 Tumor partition

**FIGURE 3:**
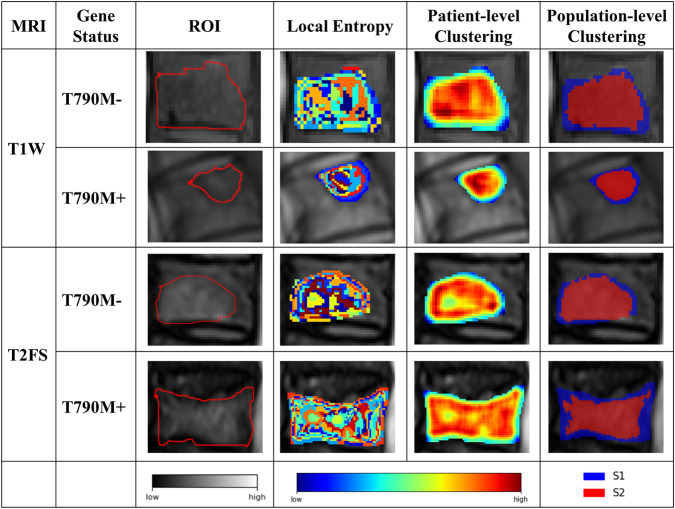
Results of the intratumor partition in the T1W and T2FS MRI. The ROI column represents the MRI images with manually segmented spinal metastases. The local entropy column represents entropy maps of the metastases.

**FIGURE 4:**
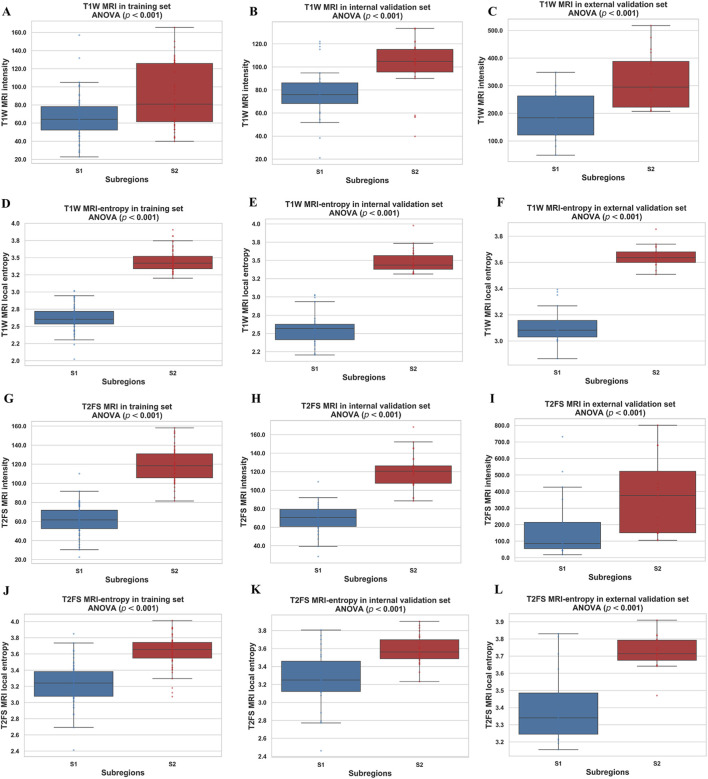
Boxplots of the MRI intensity and local entropy in the training **(A,D,G,J)**, internal validation **(B,E,H,K)**, and external validation **(C,F,I,L)** sets. Significant differences between the S1 and S2 were found by a t-test.

Based on the CH index and Silhouette coefficient (Supplementary Figure S1), the tumor was classified into two subregions with significant heterogeneity using a clustering method: marginal subregion (S1) and inner subregion (S2). Figure 3 presents the results of spinal metastases in two patients, one with wild-type EGFR and the other with an EGFR mutation. The boxplot in Figure 4 shows that S2 consistently exhibits higher MRI intensity and local entropy values compared to S1 in all patients, which may indicate a higher degree of heterogeneity in the S2 region.

### 3.3 Feature selection and model construction

The optimal features selected from the two MRI sequences were combined to identify the most important predictors. Table 3 lists the final retained features and their prediction performance, and Supplementary Table S1 provides detailed descriptions of each feature. By integrating the features from both sequences and weighting them based on LASSO regression coefficients, a multi-parametric radiomics signature was constructed, as shown in Supplementary Formula S1. After ComBat harmonization, the predictive performance of the radiomics signature and the feature values of the selected predictors are reported in Supplementary Table S2–S4.

### 3.4 Model performance across sequences and subregions

Table 2 summarizes the performance of radiomics models constructed from different tumor regions (S1, S2, and the whole tumor region) using T1W and T2FS sequences. Although the DeLong test did not show statistically significant differences (P > 0.05) in the internal and external validation cohorts, the fusion model consistently achieved higher AUCs across all datasets, indicating a consistent trend toward improved diagnostic performance. The multi-sequence regional fusion model constructed by integrating the optimal regions demonstrated enhanced predictive capability. This model achieved an AUC of 0.916 in the training set (ACC = 0.830, SPE = 0.867, SEN = 0.783), showing encouraging discriminative power. The model exhibited consistent performance, with an AUC of 0.867 (ACC = 0.815, SPE = 0.800, SEN = 0.833) on the internal validation set and an AUC of 0.839 (ACC = 0.833, SPE = 0.812, SEN = 0.857) on the external validation set. To complement discrimination with clinical utility, decision curve analysis (Supplementary Figure S2) showed that the fusion model consistently provided greater net benefit across clinically plausible threshold probabilities than all comparator models. The calibration curves (Supplementary Figure S3) indicate that the fusion model is reasonably well calibrated in all cohorts. The full set of performance metrics, including AUPRC (with 95% CI), positive predictive value (PPV), negative predictive value (NPV), and F1 score (the harmonic mean of precision and recall), are reported in Supplementary Figure S4 and Supplementary Table S5. Overall, performance patterns were generally stable across cohorts, with minor fluctuations consistent with variations in class prevalence.

**TABLE 2:** Evaluations of the RSs derived from subregions and the whole tumor regions in T1W and T2FS MRI.

**Model**	**Training**					**Internal validation**					**External validation**				
	**AUC** **(95% CI)**	**ACC**	**SPE**	**SEN**	***P***	**AUC** **(95% CI)**	**ACC**	**SPE**	**SEN**	***P***	**AUC** **(95% CI)**	**ACC**	**SPE**	**SEN**	***P***
RS-T1W-S1	0.763 (0.631 - 0.896)	0.755	0.900	0.609		0.739 (0.540 - 0.938)	0.630	0.667	0.833		0.714 (0.530 - 0.975)	0.680	0.900	0.579	
**RS-T1W-S2**	**0.817** **(0.695 - 0.938)**	**0.736**	**0.767**	**0.783**		**0.822** **(0.655 - 0.990)**	**0.778**	**0.733**	**0.833**		**0.748** **(0.568 – 0.928)**	**0.733**	**0.688**	**0.786**	
RS-T1W-W	0.808 (0.774 - 0.962)	0.736	0.633	0.913		0.801 (0.710 - 1.000)	0.704	0.667	0.886		0.708 (0.575 – 0.995)	0.727	0.762	0.688	
RS-T1W-S1 vs. RS-T1W-S2					0.162					0.454					0.414
RS-T1W-S1 vs. RS-T1W-W					0.604					0.632					0.310
RS-T1W-S2 vs. RS-T1W-W					0.352					0.556					0.560
RS-T2FS-S1	0.725 (0.582 - 0.867)	0.698	0.867	0.565		0.633 (0.393-0.874)	0.593	0.867	0.583		0.686 (0.540 - 1.000)	0.706	0.600	0.857	
**RS-T2FS-S2**	**0.817** **(0.704 - 0.931)**	**0.717**	**0.800**	**0.696**		**0.728** **(0.530 - 0.935)**	**0.778**	**0.933**	**0.583**		**0.754** **(0.576 – 0.933)**	**0.733**	**0.812**	**0.643**	
RS-T2FS-W	0.806 (0.713 - 0.979)	0.792	0.700	0.957		0.714 (0.680 - 1.000)	0.667	0.733	0.917		0.716 (0.620 - 1.000)	0.706	0.867	0.583	
RS-T2FS-S1 vs. RS-T2FS-S2					0.652					0.456					0.610
RS-T2FS-S1 vs. RS-T2FS-W					0.562					0.389					0.311
RS-T2FS-S2 vs. RS-T2FS-W					0.263					0.340					0.356
RS-W-combined	0.868 (0.774 - 0.962)	0.774	0.633	0.913		0.861 (0.710 - 1.000)	0.778	0.667	1.000		0.714 (0.571 - 1.000)	0.588	0.600	0.571	
**Multi-sequence Regional Fusion Model**	**0.916** **(0.846 - 0.986)**	**0.830**	**0.867**	**0.783**		**0.867** **(0.719 - 1.000)**	**0.815**	**0.800**	**0.833**		**0.839** **(0.690 - 0.989)**	**0.833**	**0.812**	**0.857**	
RS-T1W-S2 vs. RS-T2FS-S2					0.993					0.537					0.930
**Multi-sequence Regional Fusion Model vs. RS-T1W-S2**					0.046*					**0.616**					**0.217**
**Multi-sequence Regional Fusion Model vs. RS-T2FS-S2**					0.041*					**0.154**					**0.260**

∗*P* < 0.05

S1 subregion 1, S2 subregion 2, W whole tumor region, vs. *versus*, AUC Area Under the Receiver Operating Characteristic Curve, ACC Accuracy, SPE Specificity, SEN Sensitivity.

Bold values indicate the best-performing results within each MRI sequence and the final multi-sequence regional fusion model, and also represent the DeLong test comparisons between the best single-sequence models and the final fusion model.

### 3.5 Performance evaluation of the fusion model

**FIGURE 5:**
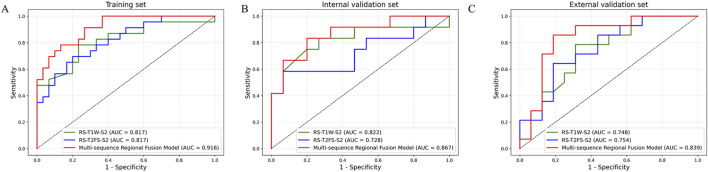
ROC curves of each RS in the training **(A)**, internal validation **(B)**, and external validation **(C)** sets.

**FIGURE 6:**
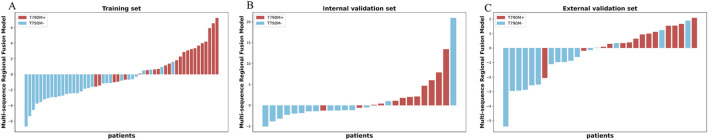
Multi-sequence Regional Fusion Model for the patients in the training **(A)**, internal validation **(B)**, and external validation **(C)** sets. The red bars indicate patients with T790M+, whereas the blue bars indicate patients with T790M-.

As shown in Figure 5, the RS-T1W-S2 and RS-T2FS-S2 exhibited consistent and comparable predictive performance. Specifically, in Figure 6, the AUC values for RS-T1W-S2 were 0.817, 0.822, and 0.748 in the training, internal, and external validation sets, respectively, while RS-T2FS-S2 achieved AUCs of 0.817, 0.728, and 0.754 in the corresponding sets. The results suggest that the diagnostic utility of single-sequence RSs remains relatively stable across different datasets. The Multi-sequence Regional Fusion Model, which integrates both T1W and T2FS sequences, consistently outperformed the single-sequence models across all datasets, yielding AUCs of 0.916 in the training set, 0.867 in the internal validation set, and 0.839 in the external validation set. The patient-level waterfall plots (Figure 6) of predicted probabilities show clearer separation between T790M+ and T790M-cases and fewer high-confidence misclassifications. Overall, these diagnostics indicate a modest but consistent advantage of the fusion model at clinically relevant decision thresholds.

### 3.6 Feature analysis

As shown in Table 3, a total of six features were identified as strongly associated with the T790M mutation following EGFR-TKI treatment, three from T1W and three from T2FS. All features demonstrated statistical significance with p-values less than 0.05 and AUC >0.65. The majority of the features (five out of six) belong to the textural feature category, which suggests that intratumoral heterogeneity is highly correlated with the T790M resistance mutation. The feature correlation heatmap for the selected features is provided in Supplementary Figure S5. To further investigate the expression patterns and consistency of these six key features across patients with different T790M mutation statuses, a hierarchical clustering heatmap was constructed, as shown in Supplementary Figure S6. Detailed explanations of these features are provided in the Supplementary Table S1 to facilitate a deeper understanding of their potential roles in the T790M mutation status.

**TABLE 3:** Performance of the selected radiomics features.

Feature	Source	Mean ± SD		AUC	*P*
		T790M+	T790M-		
log-sigma-5-0-mm-3D_gldm_DependenceNonUniformityNormalized (F1)	T1W	0.097 ± 0.037	0.065 ± 0.014	0.772	<0.001
original_glcm_InverseVariance (F2)	T1W	0.420 ± 0.042	0.452 ± 0.022	0.721	0.002
wavelet-HHH_glcm_ MCC (F3)	T1W	0.119 ± 0.028	0.094 ± 0.018	0.778	<0.001
exponential_gldm_DependenceNonUniformityNormalized (F4)	T2FS	0.151 ± 0.050	0.110 ± 0.044	0.732	0.003
lbp-3D-m2_firstorder_90Percentile (F5)	T2FS	16.856 ± 0.503	17.167 ± 0.235	0.675	0.010
log-sigma-5-0-mm-3D_glszm_SmallAreaEmphasis (F6)	T2FS	0.194 ± 0.073	0.262 ± 0.093	0.713	0.004

SD, standard deviation.

### 3.7 SHAP analysis and model interpretability

**FIGURE 7:**
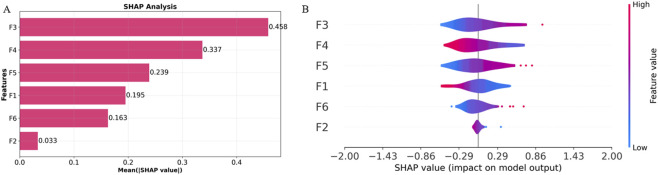
Overall visualization of the Multi-sequence Regional Fusion Model through SHAP. **(A)** The SHAP bar chart shows the weight of the six most important characteristics. **(B)** The SHAP beeswarm plot shows the positive or negative effects of each feature on the prediction probability through red and blue colors.

To enhance the interpretability and clinical applicability of the Multi-sequence Regional Fusion Model, we performed SHAP analysis to quantify the contributions of each feature. Figure 7A illustrates the mean absolute SHAP values, with F3 showing the highest value (0.458), indicating its most substantial influence on the model’s predictions, consistent with its prominent positive coefficient in the Rad-Score formula. F4 and F5 follow with SHAP values of 0.337 and 0.239, respectively, further emphasizing their significant predictive importance. In contrast, F2 exhibited the lowest SHAP value (0.033), suggesting minimal impact on the model’s predictions, which aligns with its negative coefficient in the Rad-Score formula.

The SHAP beeswarm plot in Figure 7B offers comprehensive information regarding the impact of each feature on the model’s output. F3 shows a broad SHAP value distribution, indicating that varying values of this feature significantly influence the model’s predictions, predominantly in a positive direction, which aligns with its significant positive coefficient. In contrast, F2 displays a narrow SHAP value distribution centered around zero, further confirming its minimal contribution to the model’s predictions.

Through SHAP analysis, we have clarified the specific roles of each feature within the model’s decision-making process. F3, F5, and F6 make significant contributions to the model’s predictions, while F2 and F4 have relatively minor impacts. Integrating SHAP values with the Rad-Score formula enhances the transparency of the decision-making process, improving both the model’s interpretability and its clinical applicability. These results demonstrate the value of feature selection and model interpretability, particularly in the accurate assessment of T790M mutation status.

## 4 Discussion

While MRI and other imaging techniques have advanced the molecular management of lung cancer, traditional imaging assessments often fail to capture intratumoral heterogeneity. This heterogeneity is increasingly recognized as closely linked to molecular characteristics and treatment responses (Zhang et al., 2022; Rasche et al., 2019). For patients without access to primary tumor tissue, bone metastases provide a crucial alternative for genetic evaluation. Although radiomics has been widely utilized for molecular prediction in brain metastases and other sites (Fan et al., 2023a; Cao et al., 2022; Lv et al., 2023), research specifically targeting spinal bone metastases—especially concerning the EGFR T790M resistance mutation—remains limited. To our knowledge, this study is the first to apply MRI-based radiomics to analyze intratumoral heterogeneity in spinal bone metastases and assess its clinical value in predicting T790M mutations. Additionally, our integration of model interpretability and the inclusion of data from two independent centers enhances the transparency and credibility of our findings.

Building on a robust clustering strategy based on local entropy, we objectively divided both T1W and T2FS MRI sequences into S1 and S2 for radiomics analysis. Notably, the S2 region, located in the tumor core, consistently exhibited superior predictive performance compared to the S1 region in both sequences, as reflected by higher AUCs and classification accuracies. This finding suggests that the inner subregion harbors greater biological heterogeneity and is more closely linked to the molecular status of EGFR T790M mutations, consistent with previous studies by Fan et al. (Fan et al., 2021) and Jiang et al. (Jiang et al., 2023). Biologically, the tumor core often experiences hypoxia, nutrient deprivation, and increased cellular stress (Vitale et al., 2021; Marusyk et al., 2020), which fosters the selection and expansion of cell populations with survival-promoting mutations, such as EGFR T790M alterations. Previous studies have reported that tumor bone metastases often result in bone destruction or osteolysis (Liu et al., 2022). The peripheral S1 region may correspond to the edges of destroyed bone or residual bone fragments, thus harboring less discriminative information. The inner region may be composed predominantly of metastatic tumor cells and potentially (Cao et al., 2023) contain more information associated with T790 mutation status. In contrast, marginal and fragmental regions may contain more stromal, necrotic, or inflammatory elements (Hinohara and Polyak, 2019), thus providing less specific genetic information. These results emphasize the importance of spatially resolved analysis in uncovering the molecular basis of resistance.

By integrating features from both T1W and T2FS sequences, our model consistently outperformed single-sequence models, with AUCs increasing from 0.817 to 0.916 in the training set and demonstrating similar improvements in both internal and external validation sets. This performance gain can be attributed to the complementary properties of T1W and T2FS: T1W features primarily contributed to morphological discrimination (Hanrahan and Shah, 2011), whereas T2FS features were more sensitive to water-related heterogeneity and cellularity (Lecouvet, 2016; Ruopp et al., 2008). These findings highlight the value of multi-dimensional integration for noninvasive characterization of the molecular and physiological landscape of bone metastases and support its potential clinical application in personalized molecular stratification and treatment planning. Although the DeLong test did not indicate statistical significance (P > 0.05) in the internal and external validation cohorts, this is likely due to limited sample size. Nonetheless, the fusion model consistently demonstrated higher AUCs across all datasets, supported by DCA demonstrating superior net benefit and calibration curves indicating reliable probability estimates. The fusion model exhibits a consistent trend toward better discrimination and decision net benefit; however, DeLong test in the validation cohorts is not statistically significant, and confirmation in larger, multi-center prospective studies remains necessary. Such consistency supports the added predictive value of integrating subregional features from multiple MRI sequences.

Texture features, as pivotal components of radiomics, provide quantitative descriptors of spatial complexity and gray-level heterogeneity within tumors. Increasing evidence has demonstrated that these features serve as noninvasive imaging surrogates for intratumoral biological diversity and therapy resistance (Gillies et al., 2016; Aerts et al., 2014). For example, Aerts et al. systematically revealed that textural heterogeneity features are associated with underlying gene-expression patterns and proliferative activity (Aerts et al., 2014). These findings highlight the essential role of spatial heterogeneity, captured by radiomic texture features, in reflecting the clonal diversity and microenvironmental adaptation of tumors under therapeutic pressure. In this study, six radiomic features were identified from the S2 subregion—three from each of the T1W and T2FS sequences—that showed strong associations with the T790M mutation status. We found that five of these features were textural, and all exhibited consistent predictive performance (*P* < 0.05, AUC >0.65). This illustrates the important function of spatial heterogeneity, as captured by texture features, in the emergence of acquired resistance. These results reinforce the paradigm that intratumoral textural heterogeneity, as quantified by radiomics, serves as a critical imaging biomarker for drug resistance, likely reflecting underlying clonal diversity and adaptive microenvironmental changes in metastatic lesions.

At the microscopic level, the most influential features, F1 (DependenceNonUniformityNormalized, GLDM), F6 (SmallAreaEmphasis, GLSZM), and F3 (MCC, GLCM), capture complementary aspects of tumor biology that are closely linked to acquired resistance. Specifically, DependenceNonUniformityNormalized quantifies the irregularity of local gray-level dependencies, may reflecting increased cellular heterogeneity, disorganized tissue structure, or the coexistence of proliferative and necrotic areas, hallmarks of aggressive, therapy-resistant. SmallAreaEmphasis highlights the presence of small, high-intensity regions, which may correspond to clusters of densely packed tumor cells or focal necrosis, indicative of microenvironmental adaptation and clonal selection under therapeutic pressure. MCC measures the spatial linear complexity of intensity distributions, serving as a putative marker for underlying genetic and cellular diversity. Collectively, these features act as noninvasive imaging proxies for complex pathological processes, such as cellular heterogeneity, necrosis, and architectural disorganization, that drive the emergence of aggressive, drug-resistant tumor phenotypes. Collectively, these texture features are theoretically well aligned with the clonal selection mechanism that drives EGFR T790M mutation. T790M is one of the most common mechanisms of acquired resistance to first- or second-generation EGFR-TKIs; its essence is the expansion of resistant subclones under therapeutic pressure (Qi et al., 2024; Dong et al., 2024; O'Connor et al., 2015), which is biologically manifested as intratumoral heterogeneity (including variation in cell density, coexistence of necrotic and viable regions, and intermixing of dying vs. surviving cells). Secondly, such texture features represent whole-tumor heterogeneity, whereas biopsies usually sample only a local region and thus may miss resistant subregions (Li and Zhou, 2022; Frankell et al., 2023; Gerlinger et al., 2012). However, we also explicitly acknowledge that biological interpretability remains limited.

A significant difference in smoking status was found between T790M-positive and T790M-negative patients (45.8% vs. 10.3%, P = 0.009) in the training set. Two previous studies demonstrated that smoking was significantly associated with T790M mutation status (P < 0.05) (Ke et al., 2017; Jaiswal et al., 2019), which is consistent with our findings. This association may be explained by the fact that smoking increases tumor mutational burden and subclonal diversity, thereby facilitating resistance to tumor therapy (Blakely et al., 2017; Frankell et al., 2023). However, smoking remains a controversial factor, as some previous studies reported no significant association (P > 0.05) (Huang et al., 2018; Wu et al., 2024; Lv et al., 2023; Tseng et al., 2016; Ouyang et al., 2020).

SHAP analysis confirmed the dominant contributions of F3 (MCC), F4 (DependenceNonUniformityNormalized), and F5 (firstorder 90Percentile) to model predictions, consistent with their prominent positive coefficients in the Rad-Score formula. In contrast, features such as InverseVariance (F2) had minimal impact, underscoring the specificity and biological relevance of the key textural predictors. By elucidating the internal decision logic of the model, SHAP analysis enhances interpretability and transparency, enabling clinicians to better trust and adopt radiomics-based tools in precision oncology. In multicenter MRI radiomics, scanner variability poses a major challenge. In our study, ComBat harmonization failed to improve predictive performance, consistent with prior work (Li et al., 2021; Zhang et al., 2024a; Ibrahim et al., 2022). Therefore, we instead employed multistep preprocessing and ICC-based filtering to enhance robustness.

This study has several limitations. First, this retrospective analysis has a limited sample size, particularly in the external validation set, which creates potential for selection bias. Further studies should be conducted with larger sample sizes. Second, although the reliability and validity of the segmentation approach have been previously demonstrated (Fan et al., 2021; Hong et al., 2020), it may not fully capture the microstructural heterogeneity of bone metastases. Third, the stability of radiomic features is dependent on image quality and may be influenced by artifacts or scanner variability. Fourth, this study only analyzed T1W and T2FS MRI sequences and did not incorporate functional imaging (e.g., T1CE, DWI) or multi-omics data, which may limit the completeness of feature representation and model generalizability. Finally, although SHAP analysis provided only a preliminary level of model interpretability, the identified MRI features still lack biological validation, and their associations with tumor biology remain unclear.

## 5 Conclusion

This study demonstrates that MRI-based radiomics can effectively and non-invasively predict EGFR T790M mutations in spinal metastases. Model performance was improved by integrating features from S2 and combining T1W and T2FS sequences. Its potential as a noninvasive tool for guiding EGFR-TKI therapy in NSCLC patients with inaccessible spinal metastases.

## Data Availability

The original contributions presented in the study are included in the article/Supplementary Material, further inquiries can be directed to the corresponding author.

## References

[B1] Aerts H. J. Velazquez E. R. Leijenaar R. T. Parmar C. Grossmann P. Carvalho S. (2014). Decoding tumour phenotype by noninvasive imaging using a quantitative radiomics approach. Nat. Commun. 5, 4006. 10.1038/ncomms5006 24892406 PMC4059926

[B2] Blakely C. M. Watkins T. B. Wu W. Gini B. Chabon J. J. Mccoach C. E. (2017). Evolution and clinical impact of co-occurring genetic alterations in advanced-stage EGFR-Mutant lung cancers. Nat. Genet. 49, 1693–1704. 10.1038/ng.3990 29106415 PMC5709185

[B3] Brabec J. KomáREK T. Franc V. Machlica L. (2020). “On model evaluation under non-constant class imbalance,” in International conference on computational science. Springer, 74–87.

[B4] Caliński T. Harabasz J. (1974). A dendrite method for cluster analysis. Commun. Statistics-theory Methods 3, 1–27. 10.1080/03610927408827101

[B5] Cao R. Pang Z. Wang X. DU Z. Chen H. Liu J. (2022). Radiomics evaluates the EGFR mutation status from the brain metastasis: a multi-center study. Phys. Med. and Biol. 67, 125003. 10.1088/1361-6560/ac7192 35588722

[B6] Cao R. Chen H. Wang H. Wang Y. Cui E.-N. Jiang W. (2023). Comprehensive analysis of prediction of the EGFR mutation and subtypes based on the spinal metastasis from primary lung adenocarcinoma. Front. Oncol. 13, 1154327. 10.3389/fonc.2023.1154327 37143947 PMC10151709

[B7] CléZARDIN P. Coleman R. Puppo M. Ottewell P. Bonnelye E. Paycha F. (2021). Bone metastasis: mechanisms, therapies, and biomarkers. Physiol. Rev. 101, 797–855. 10.1152/physrev.00012.2019 33356915

[B8] Coleman R. (2001). Metastatic bone disease: clinical features, pathophysiology and treatment strategies. Cancer Treat. Rev. 27, 165–176. 10.1053/ctrv.2000.0210 11417967

[B9] Cross D. A. Ashton S. E. Ghiorghiu S. Eberlein C. Nebhan C. A. Spitzler P. J. (2014). AZD9291, an irreversible EGFR TKI, overcomes T790M-mediated resistance to EGFR inhibitors in lung cancer. Cancer Discov. 4, 1046–1061. 10.1158/2159-8290.CD-14-0337 24893891 PMC4315625

[B10] Dagogo-Jack I. Shaw A. T. (2018). Tumour heterogeneity and resistance to cancer therapies. Nat. Rev. Clin. Oncol. 15, 81–94. 10.1038/nrclinonc.2017.166 29115304

[B11] Demircioğlu A. (2021). Measuring the bias of incorrect application of feature selection when using cross-validation in radiomics. Insights into Imaging 12, 172. 10.1186/s13244-021-01115-1 34817740 PMC8613324

[B12] Dong X. Chen G. Zhu Y. Ma B. Ban X. Wu N. (2024). Artificial intelligence in skeletal metastasis imaging. Comput. Struct. Biotechnol. J. 23, 157–164. 10.1016/j.csbj.2023.11.007 38144945 PMC10749216

[B13] Fan Y. Dong Y. Yang H. Chen H. Yu Y. Wang X. (2021). Subregional radiomics analysis for the detection of the EGFR mutation on thoracic spinal metastases from lung cancer. Phys. Med. and Biol. 66, 215008. 10.1088/1361-6560/ac2ea7 34633298

[B14] Fan Y. He L. Yang H. Wang Y. Su J. Hou S. (2023a). Preoperative MRI‐based radiomics of brain metastasis to assess T790M resistance mutation after EGFR‐TKI treatment in NSCLC. J. Magnetic Reson. Imaging 57, 1778–1787. 10.1002/jmri.28441 36165534

[B15] Fan Y. Wang X. Yang C. Chen H. Wang H. Wang X. (2023b). Brain‐tumor interface‐based MRI radiomics models to determine EGFR mutation, response to EGFR‐TKI and T790M resistance mutation in non‐small cell lung carcinoma brain metastasis. J. Magnetic Reson. Imaging 58, 1838–1847. 10.1002/jmri.28751 37144750

[B16] Frankell A. M. Dietzen M. Al Bakir M. Lim E. L. Karasaki T. Ward S. (2023). The evolution of lung cancer and impact of subclonal selection in TRACERx. Nature 616, 525–533. 10.1038/s41586-023-05783-5 37046096 PMC10115649

[B17] Gerlinger M. Rowan A. J. Horswell S. Larkin J. Endesfelder D. Gronroos E. (2012). Intratumor heterogeneity and branched evolution revealed by multiregion sequencing. N. Engl. J. Med. 366, 883–892. 10.1056/NEJMoa1113205 22397650 PMC4878653

[B18] Gillies R. J. Kinahan P. E. Hricak H. (2016). Radiomics: images are more than pictures, they are data. Radiology 278, 563–577. 10.1148/radiol.2015151169 26579733 PMC4734157

[B19] Gutman D. A. Cooper L. A. Hwang S. N. Holder C. A. Gao J. Aurora T. D. (2013). MR imaging predictors of molecular profile and survival: multi-institutional study of the TCGA glioblastoma data set. Radiology 267, 560–569. 10.1148/radiol.13120118 23392431 PMC3632807

[B20] Hanrahan C. J. Shah L. M. (2011). MRI of spinal bone marrow: part 2, T1-weighted imaging-based differential diagnosis. Am. J. Roentgenol. 197, 1309–1321. 10.2214/AJR.11.7420 22109284

[B21] Hinohara K. Polyak K. (2019). Intratumoral heterogeneity: more than just mutations. Trends Cell Biol. 29, 569–579. 10.1016/j.tcb.2019.03.003 30987806 PMC6579620

[B22] Hong D. Xu K. Zhang L. Wan X. Guo Y. (2020). Radiomics signature as a predictive factor for EGFR mutations in advanced lung adenocarcinoma. Front. Oncol. 10, 28. 10.3389/fonc.2020.00028 32082997 PMC7005234

[B23] Huang Y.-H. Hsu K.-H. Tseng J.-S. Chen K.-C. Hsu C.-H. Su K.-Y. (2018). The association of acquired T790M mutation with clinical characteristics after resistance to first-line epidermal growth factor receptor tyrosine kinase inhibitor in lung adenocarcinoma. Cancer Res. Treat. official J. Korean Cancer Assoc. 50, 1294–1303. 10.4143/crt.2017.512 29334606 PMC6192936

[B24] Ibrahim A. Primakov S. Beuque M. Woodruff H. Halilaj I. Wu G. (2021). Radiomics for precision medicine: current challenges, future prospects, and the proposal of a new framework. Methods 188, 20–29. 10.1016/j.ymeth.2020.05.022 32504782

[B25] Ibrahim A. Lu L. Yang H. Akin O. Schwartz L. H. Zhao B. (2022). The impact of image acquisition parameters and ComBat harmonization on the predictive performance of radiomics: a renal cell carcinoma model. Appl. Sci. 12, 9824. 10.3390/app12199824 37091743 PMC10121203

[B26] Jaiswal R. Pinninti R. Mohan M. K. Santa A. Boyella P. K. Nambaru L. (2019). T790M mutation and clinical outcomes with osimertinib in patients with epidermal growth factor receptor-mutant nonsmall cell lung cancer. Indian J. Med. Paediatr. Oncol. 40, 73–78. 10.4103/ijmpo.ijmpo_215_18

[B27] Jiang T. Sun X. Dong Y. Guo W. Wang H. Yue Z. (2023). Deep learning for preoperative prediction of the EGFR mutation and subtypes based on the MRI image of spinal metastasis from primary NSCLC. Biomed. Signal Process. Control 79, 104084. 10.1016/j.bspc.2022.104084

[B28] Ke E. Zhou Q. Zhang Q.-Y. Su J. Chen Z.-H. Zhang X.-C. (2017). A higher proportion of the EGFR T790M mutation May contribute to the better survival of patients with exon 19 deletions compared with those with L858R. J. Thorac. Oncol. 12, 1368–1375. 10.1016/j.jtho.2017.05.018 28576746

[B29] Kobayashi K. Tan A. C. (2023). Unraveling the impact of intratumoral heterogeneity on EGFR tyrosine kinase inhibitor resistance in EGFR-Mutated NSCLC. Int. J. Mol. Sci. 24, 4126. 10.3390/ijms24044126 36835536 PMC9964908

[B30] Lambin P. Leijenaar R. T. Deist T. M. Peerlings J. De Jong E. E. Van Timmeren J. (2017). Radiomics: the bridge between medical imaging and personalized medicine. Nat. Rev. Clin. Oncol. 14, 749–762. 10.1038/nrclinonc.2017.141 28975929

[B31] Lecouvet F. E. (2016). Whole-body MR imaging: musculoskeletal applications. Radiology 279, 345–365. 10.1148/radiol.2016142084 27089188

[B32] Li S. Zhou B. (2022). A review of radiomics and genomics applications in cancers: the way towards precision medicine. Radiat. Oncol. 17, 217. 10.1186/s13014-022-02192-2 36585716 PMC9801589

[B33] Li Y. Ammari S. Balleyguier C. Lassau N. Chouzenoux E. (2021). Impact of preprocessing and harmonization methods on the removal of scanner effects in brain MRI radiomic features. Cancers 13, 3000. 10.3390/cancers13123000 34203896 PMC8232807

[B34] Li Y. Lv X. Wang Y. Xu Z. Lv Y. Hou D. (2023). CT-based nomogram for early identification of T790M resistance in metastatic non-small cell lung cancer before first-line epidermal growth factor receptor-tyrosine kinase inhibitors therapy. Eur. Radiol. Exp. 7, 64. 10.1186/s41747-023-00380-7 37914925 PMC10620367

[B35] Lim Z.-F. Ma P. C. (2019). Emerging insights of tumor heterogeneity and drug resistance mechanisms in lung cancer targeted therapy. J. Hematol. and Oncol. 12, 134. 10.1186/s13045-019-0818-2 31815659 PMC6902404

[B36] Liu K. Zhang Y. Wang Q. Chen Y. Qin S. Xin P. (2022). Differentiation of predominantly osteolytic from osteoblastic spinal metastases based on standard magnetic resonance imaging sequences: a comparison of radiomics model *versus* semantic features logistic regression model findings. Quantitative Imaging Med. Surg. 12, 5004–5017. 10.21037/qims-22-267 36330195 PMC9622449

[B37] Lu J. Ji X. Liu X. Jiang Y. Li G. Fang P. (2024). Machine learning-based radiomics strategy for prediction of acquired EGFR T790M mutation following treatment with EGFR-TKI in NSCLC. Sci. Rep. 14, 446. 10.1038/s41598-023-50984-7 38172228 PMC10764785

[B38] Lv X. Li Y. Wang B. Wang Y. Pan Y. Li C. (2023). Multisequence MRI-Based radiomics analysis for early prediction of the risk of T790M resistance in new brain metastases. Quantitative Imaging Med. Surg. 13, 8599–8610. 10.21037/qims-23-822 38106277 PMC10722019

[B39] Marusyk A. Janiszewska M. Polyak K. (2020). Intratumor heterogeneity: the rosetta stone of therapy resistance. Cancer Cell 37, 471–484. 10.1016/j.ccell.2020.03.007 32289271 PMC7181408

[B40] Mcgranahan N. Swanton C. (2017). Clonal heterogeneity and tumor evolution: past, present, and the future. Cell 168, 613–628. 10.1016/j.cell.2017.01.018 28187284

[B41] Mok T. S. Wu Y.-L. Ahn M.-J. Garassino M. C. Kim H. R. Ramalingam S. S. (2017). Osimertinib or platinum–pemetrexed in EGFR T790M–positive lung cancer. N. Engl. J. Med. 376, 629–640. 10.1056/NEJMoa1612674 27959700 PMC6762027

[B42] O'Connor J. P. Rose C. J. Waterton J. C. Carano R. A. Parker G. J. Jackson A. (2015). Imaging intratumor heterogeneity: role in therapy response, resistance, and clinical outcome. Clin. Cancer Res. 21, 249–257. 10.1158/1078-0432.CCR-14-0990 25421725 PMC4688961

[B43] Ouyang W. Yu J. Huang Z. Chen G. Liu Y. Liao Z. (2020). Risk factors of acquired T790M mutation in patients with epidermal growth factor receptor-mutated advanced non-small cell lung cancer. J. Cancer 11, 2060–2067. 10.7150/jca.37991 32127933 PMC7052924

[B44] Pao W. Miller V. A. Politi K. A. Riely G. J. Somwar R. Zakowski M. F. (2005). Acquired resistance of lung adenocarcinomas to gefitinib or erlotinib is associated with a second mutation in the EGFR kinase domain. PLoS Med. 2, e73. 10.1371/journal.pmed.0020073 15737014 PMC549606

[B45] Pham D. T. Dimov S. S. Nguyen C. D. (2005). Selection of K in K-means clustering. Proc. Institution Mech. Eng. Part C J. Mech. Eng. Sci. 219, 103–119. 10.1243/095440605X8298

[B46] Qi H. Hou Y. Zheng Z. Zheng M. Qiao Q. Wang Z. (2024). Clinical characteristics and MRI based radiomics nomograms can predict iPFS and short-term efficacy of third-generation EGFR-TKI in EGFR-Mutated lung adenocarcinoma with brain metastases. BMC Cancer 24, 362. 10.1186/s12885-024-12121-z 38515096 PMC10956298

[B47] Ramalingam S. S. Vansteenkiste J. Planchard D. Cho B. C. Gray J. E. Ohe Y. (2020). Overall survival with osimertinib in untreated, EGFR-Mutated advanced NSCLC. N. Engl. J. Med. 382, 41–50. 10.1056/NEJMoa1913662 31751012

[B48] Rasche L. KortüM K. M. Raab M. S. Weinhold N. (2019). The impact of tumor heterogeneity on diagnostics and novel therapeutic strategies in multiple myeloma. Int. J. Mol. Sci. 20, 1248. 10.3390/ijms20051248 30871078 PMC6429294

[B49] Rolfo C. Mack P. Scagliotti G. V. Aggarwal C. Arcila M. E. Barlesi F. (2021). Liquid biopsy for advanced NSCLC: a consensus statement from the international association for the study of lung cancer. J. Thorac. Oncol. 16, 1647–1662. 10.1016/j.jtho.2021.06.017 34246791

[B50] Ruopp M. D. Perkins N. J. Whitcomb B. W. Schisterman E. F. (2008). Youden index and optimal cut‐point estimated from observations affected by a lower limit of detection. Biometrical J. J. Math. Methods Biosci. 50, 419–430. 10.1002/bimj.200710415 18435502 PMC2515362

[B51] Shi S. Wang H. Liu X. Xiao J. (2021). Prediction of overall survival of non-small cell lung cancer with bone metastasis: an analysis of the surveillance, epidemiology and end results (SEER) database. Transl. Cancer Res. 10, 5191–5203. 10.21037/tcr-21-1507 35116369 PMC8797363

[B52] Shirley M. Keam S. J. (2022). Aumolertinib: a review in non-small cell lung cancer. Drugs 82, 577–584. 10.1007/s40265-022-01695-2 35305259

[B53] Siravegna G. Marsoni S. Siena S. Bardelli A. (2017). Integrating liquid biopsies into the management of cancer. Nat. Rev. Clin. Oncol. 14, 531–548. 10.1038/nrclinonc.2017.14 28252003

[B54] Soria J.-C. Ohe Y. Vansteenkiste J. Reungwetwattana T. Chewaskulyong B. Lee K. H. (2018). Osimertinib in untreated EGFR-Mutated advanced non–small-cell lung cancer. N. Engl. J. Med. 378, 113–125. 10.1056/NEJMoa1713137 29151359

[B55] Sung H. Ferlay J. Siegel R. L. Laversanne M. Soerjomataram I. Jemal A. (2021). Global cancer statistics 2020: GLOBOCAN estimates of incidence and mortality worldwide for 36 cancers in 185 countries. CA a cancer J. Clin. 71, 209–249. 10.3322/caac.21660 33538338

[B56] Thai A. A. Solomon B. J. Sequist L. V. Gainor J. F. Heist R. S. (2021). Lung cancer. Lancet 398, 535–554. 10.1016/s0140-6736(21)00312-3 34273294

[B57] Tseng J.-S. Su K.-Y. Yang T.-Y. Chen K.-C. Hsu K.-H. Chen H.-Y. (2016). The emergence of T790M mutation in EGFR-Mutant lung adenocarcinoma patients having a history of acquired resistance to EGFR-TKI: focus on rebiopsy timing and long-term existence of T790M. Oncotarget 7, 48059–48069. 10.18632/oncotarget.10351 27384480 PMC5217000

[B58] Van Calster B. Mclernon D. J. Van Smeden M. Wynants L. Steyerberg E. W. Tests T. G. E. D. (2019). Calibration: the achilles heel of predictive analytics. BMC Med. 17, 230. 10.1186/s12916-019-1466-7 31842878 PMC6912996

[B59] Vickers A. J. Elkin E. B. (2006). Decision curve analysis: a novel method for evaluating prediction models. Med. Decis. Mak. 26, 565–574. 10.1177/0272989X06295361 17099194 PMC2577036

[B60] Vitale I. Shema E. Loi S. Galluzzi L. (2021). Intratumoral heterogeneity in cancer progression and response to immunotherapy. Nat. Med. 27, 212–224. 10.1038/s41591-021-01233-9 33574607

[B61] Wang Y. Yu W. Shi J. Qiu R. Jiang N. Wang Z. (2022). Evaluating the efficacy of EGFR-TKIs combined with radiotherapy in advanced lung adenocarcinoma patients with EGFR mutation: a retrospective study. Technol. Cancer Res. and Treat. 21, 15330338221100358. 10.1177/15330338221100358 35607295 PMC9134423

[B62] Wu S. Pan Y. Mao Y. Chen Y. He Y. (2021). Current progress and mechanisms of bone metastasis in lung cancer: a narrative review. Transl. Lung Cancer Res. 10, 439–451. 10.21037/tlcr-20-835 33569325 PMC7867745

[B63] Wu W.-F. Lai K.-M. Chen C.-H. Wang B.-C. Chen Y.-J. Shen C.-W. (2024). Predicting the T790M mutation in non-small cell lung cancer (NSCLC) using brain metastasis MR radiomics: a study with an imbalanced dataset. Discov. Oncol. 15, 447. 10.1007/s12672-024-01333-1 39277568 PMC11401825

[B64] Zhang A. Miao K. Sun H. Deng C.-X. (2022). Tumor heterogeneity reshapes the tumor microenvironment to influence drug resistance. Int. J. Biol. Sci. 18, 3019–3033. 10.7150/ijbs.72534 35541919 PMC9066118

[B65] Zhang X. Iqbal Bin Saripan M. Wu Y. Wang Z. Wen D. Cao Z. (2024a). The impact of the combat method on radiomics feature compensation and analysis of scanners from different manufacturers. BMC Med. Imaging 24, 137. 10.1186/s12880-024-01306-4 38844854 PMC11157873

[B66] Zhang X. Zhang G. Qiu X. Yin J. Tan W. Yin X. (2024b). Exploring non-invasive precision treatment in non-small cell lung cancer patients through deep learning radiomics across imaging features and molecular phenotypes. Biomark. Res. 12, 12. 10.1186/s40364-024-00561-5 38273398 PMC10809593

