# Supplementary figures and images for: MRI-based radiomics for noninvasive prediction of T790M resistance mutation in lung cancer spinal metastases: an exploratory study
Source: Front Cell Dev Biol. 2025 Oct 29;13:1673498. doi: 10.3389/fcell.2025.1673498 (PMC12605000; doi:10.3389/fcell.2025.1673498)

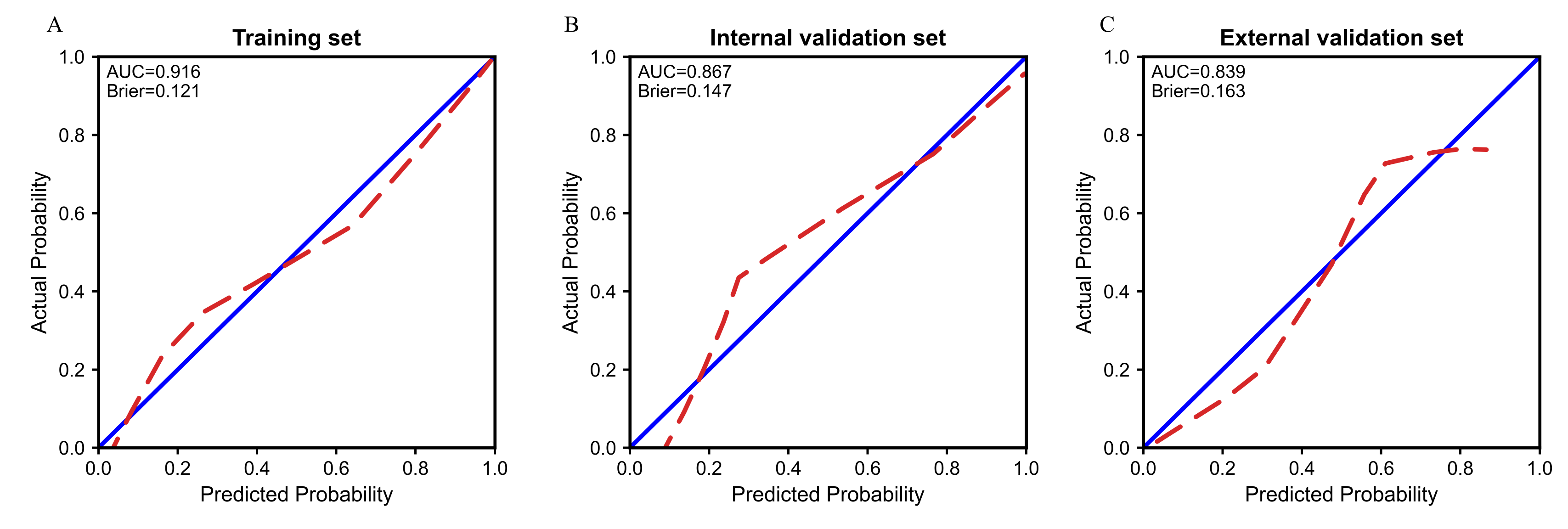

Supplement: Supplementary file 1 [file Image3.tif]

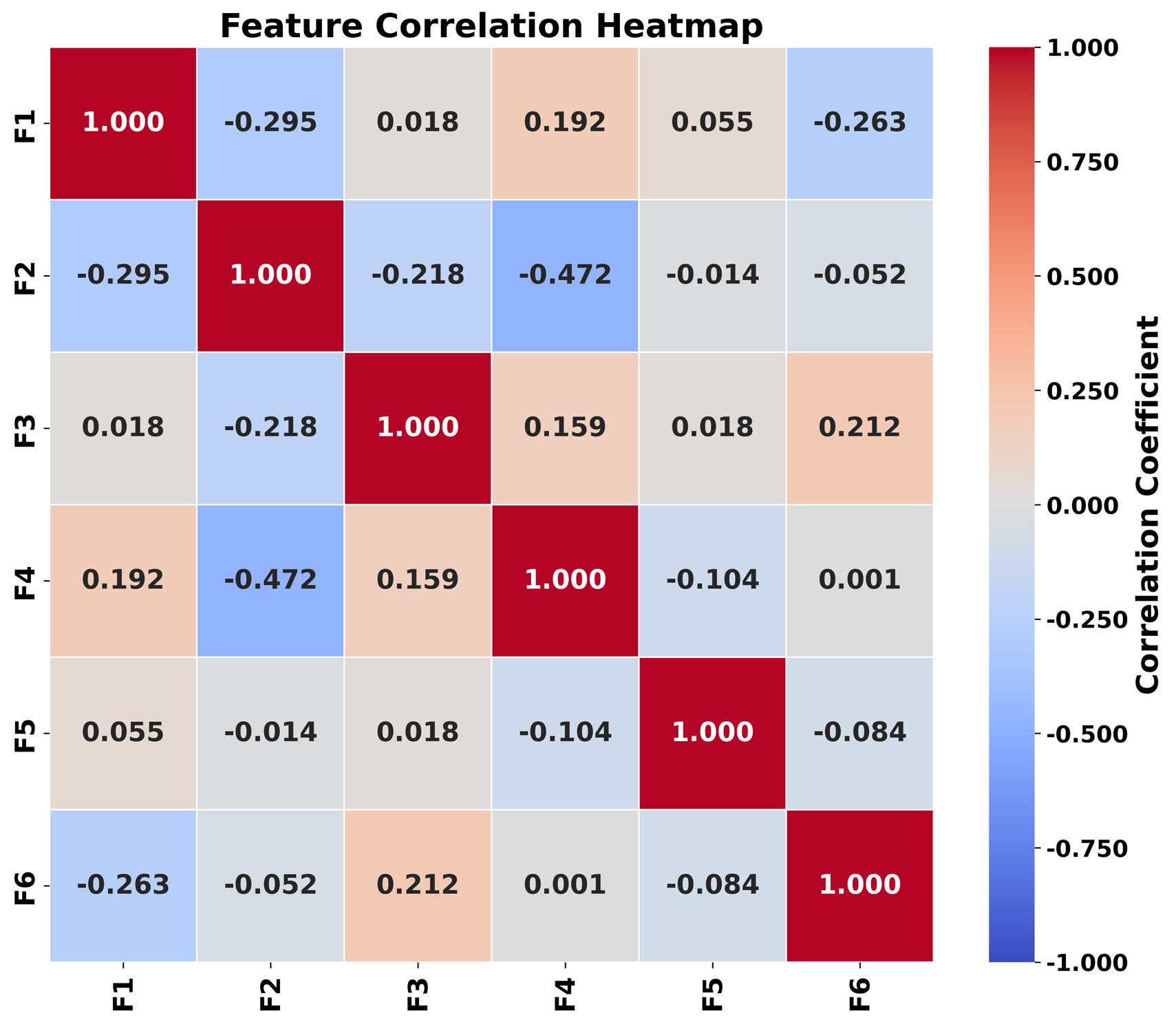

Supplement: Supplementary file 2 [file Image5.tiff]

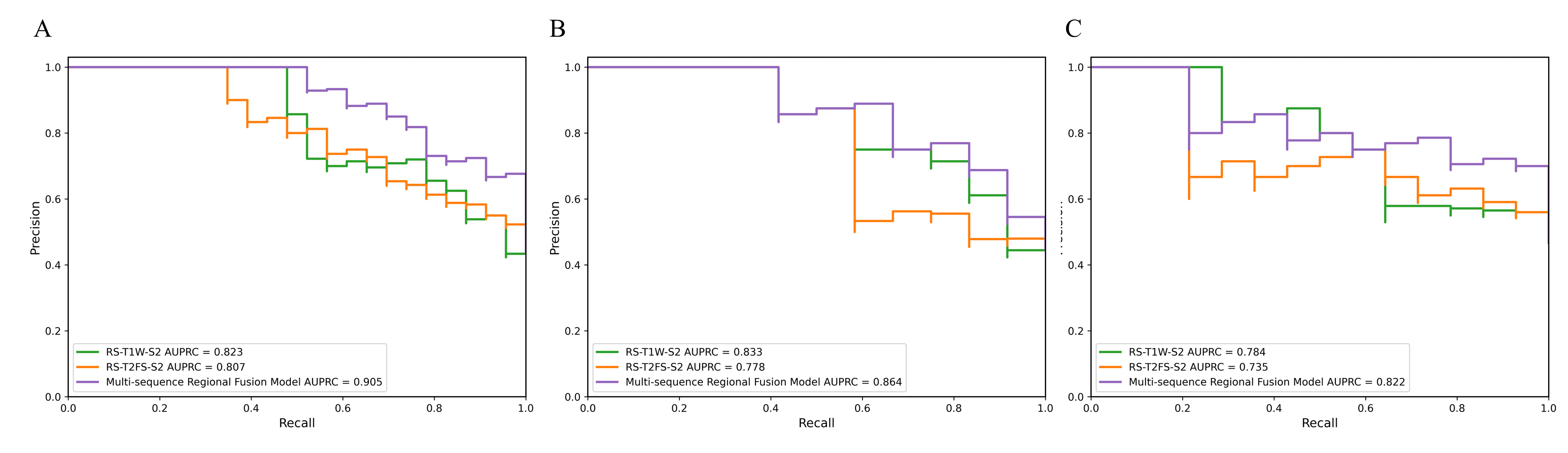

Supplement: Supplementary file 3 [file Image4.tif]

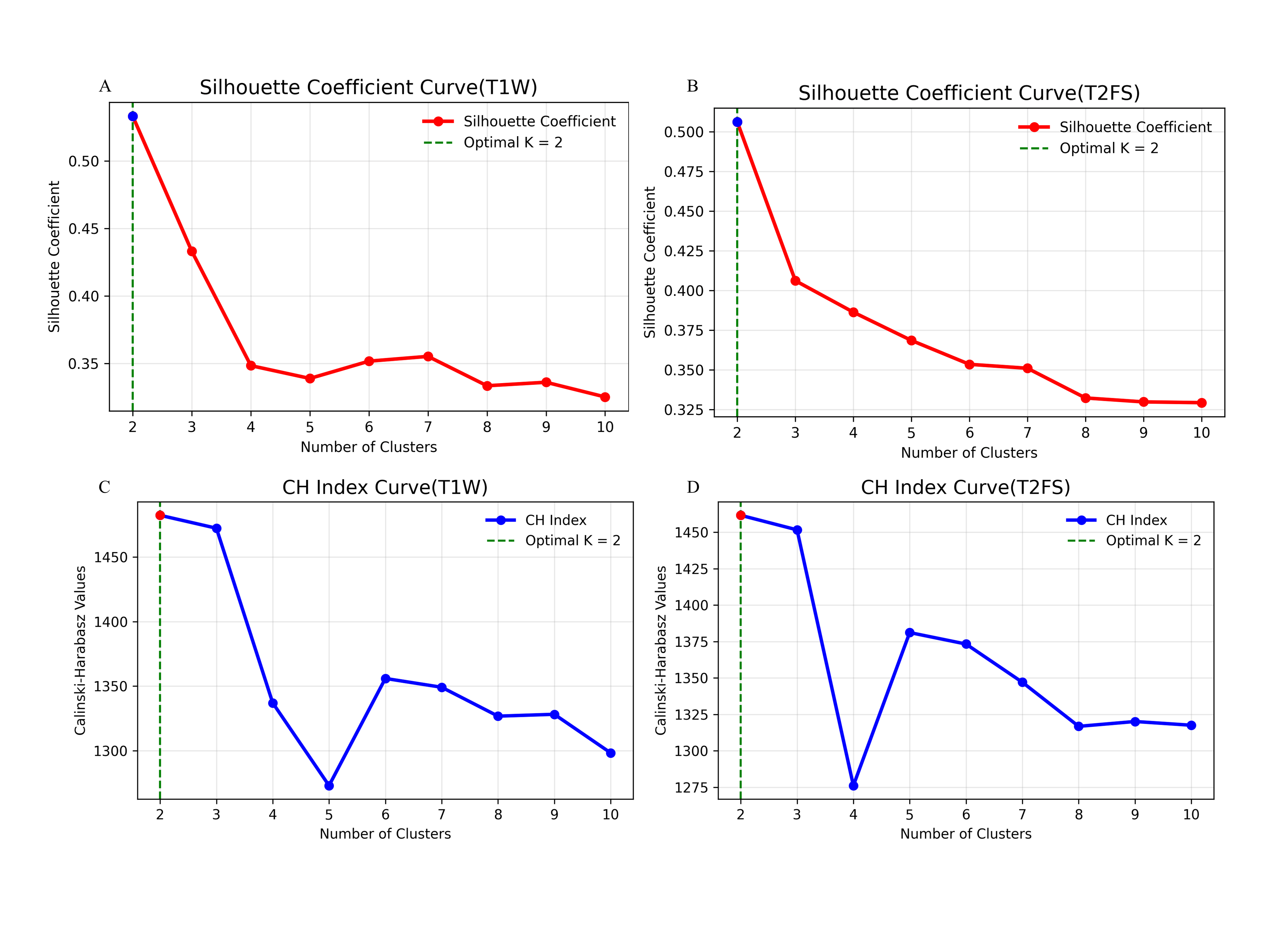

Supplement: Supplementary file 4 [file Image1.tif]

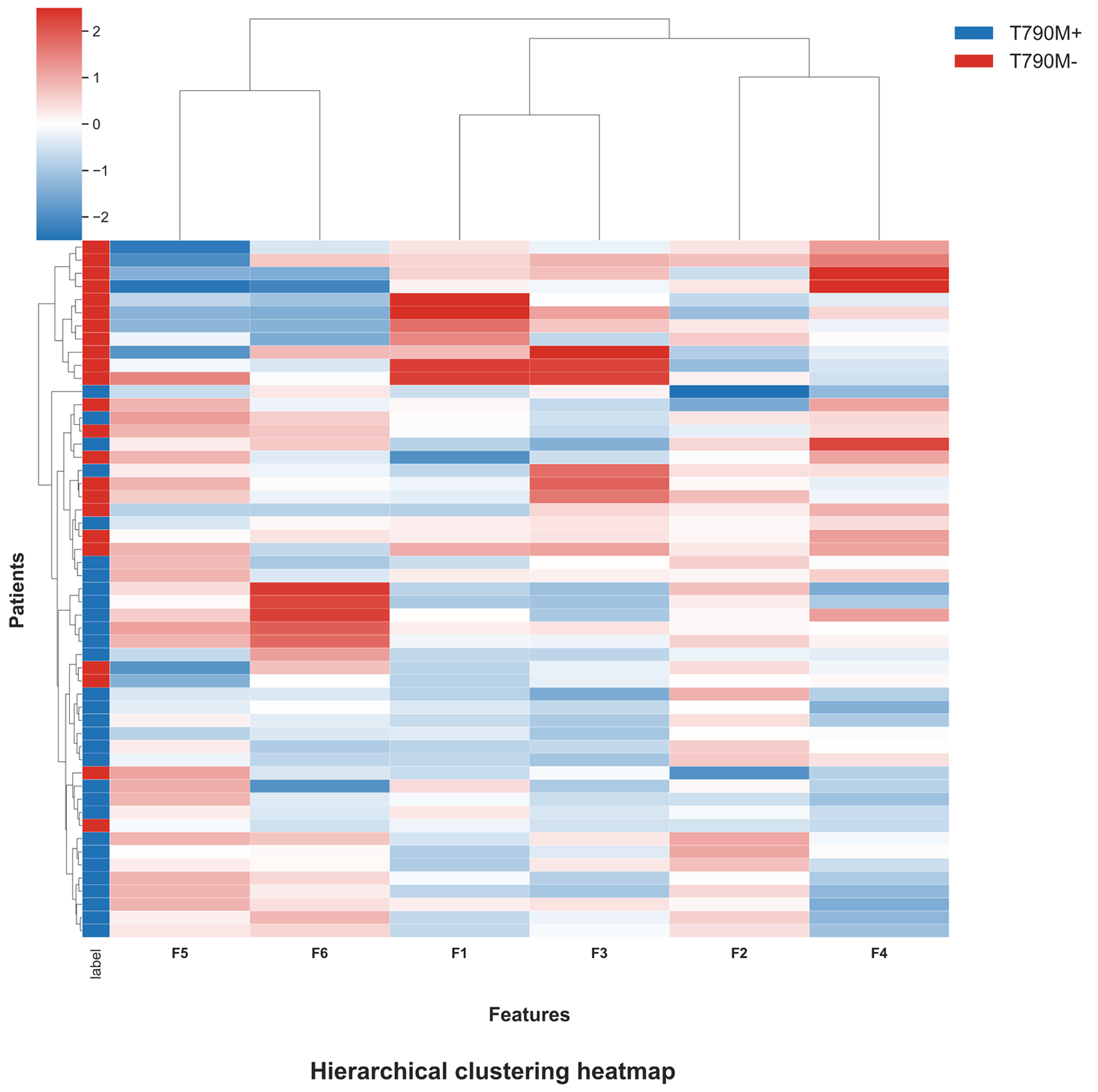

Supplement: Supplementary file 6 [file Image6.tiff]

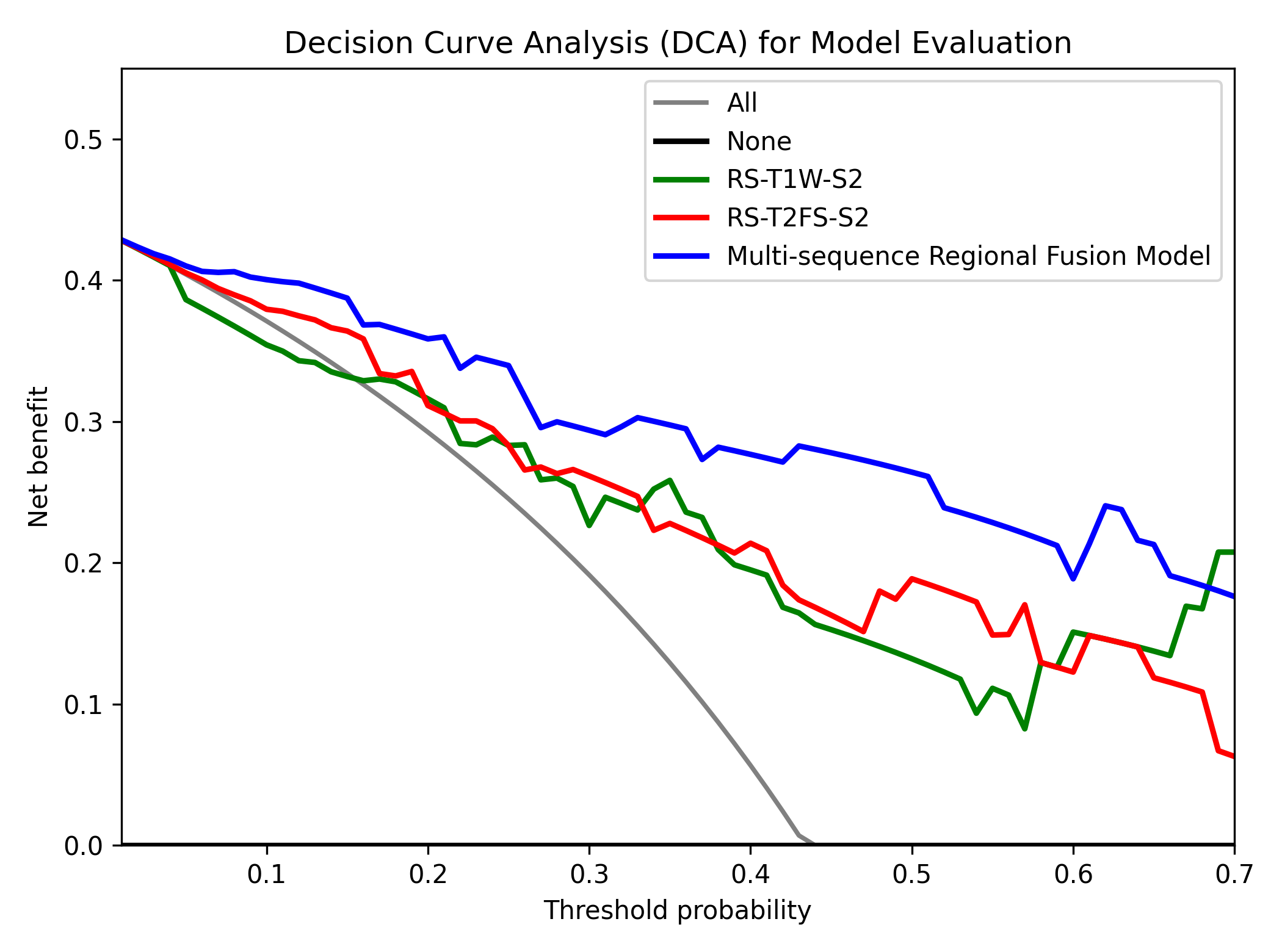

Supplement: Supplementary file 7 [file Image2.tiff]
