# Supplementary material for: MRI-based radiomics for noninvasive prediction of T790M resistance mutation in lung cancer spinal metastases: an exploratory study
Source: Front Cell Dev Biol. 2025 Oct 29;13:1673498. doi: 10.3389/fcell.2025.1673498 (PMC12605000; doi:10.3389/fcell.2025.1673498)
Supplement: Supplementary file 5 [file DataSheet1.pdf]

Supplementary Material

Table of Contents

Supplementary Figure S1	1
Supplementary Figure S2	2
Supplementary Figure S3	2
Supplementary Figure S4	3
Supplementary Figure S5	3
Supplementary Figure S6	4
Supplementary Method 1	5
Supplementary Formula S1	7
Supplementary Table S1	8
Supplementary Table S2	9
Supplementary Table S3	10
Supplementary Table S4	11
Supplementary Table S5	12

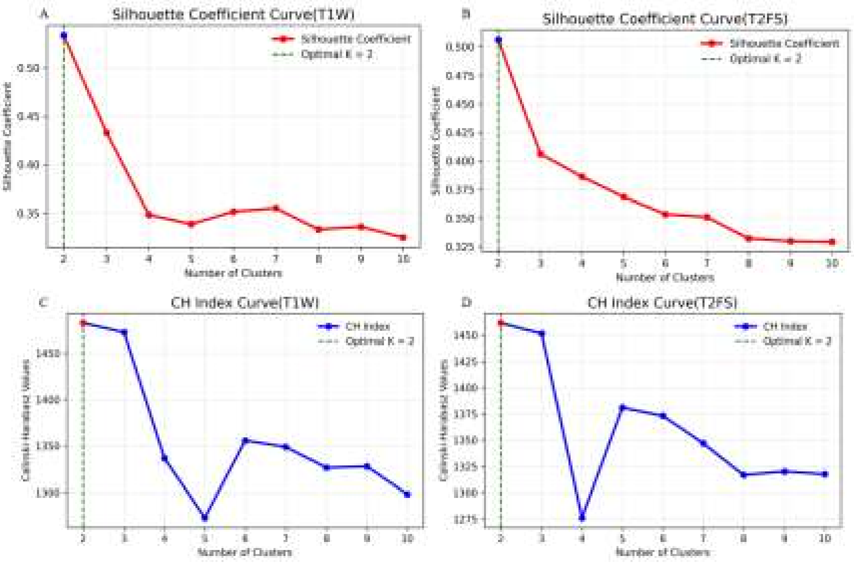

Supplementary Figure S1. Determination of optimal cluster number for K-means. The optimal number of clusters was determined using the Silhouette Coefficient and Calinski-Harabasz Index curves for T1W (A, C) and T2FS (B, D) MRI images.

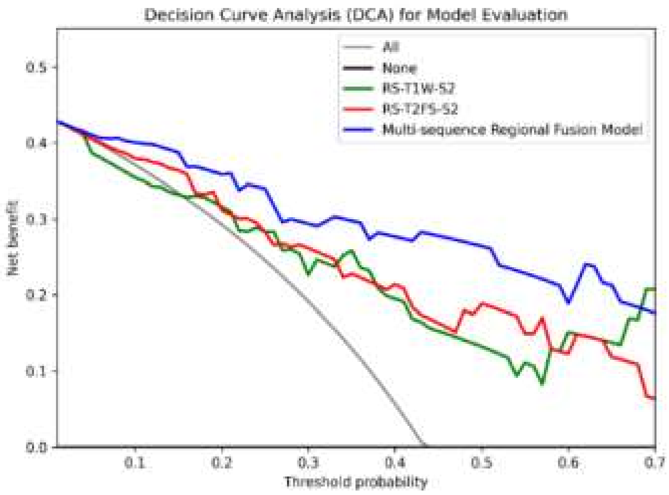

Supplementary Figure S2. Decision Curve Analysis (DCA) for Model Evaluation. This plot illustrates the net benefit of various models (RS-T1W-S2, RS-T2FS-S2, and the Multi-sequence Regional Fusion Model) across a range of threshold probabilities. For comparison, the net benefits of the "All" and "None" strategies are also presented. The Multi-sequence Regional Fusion Model consistently achieves the highest net benefit, especially at higher threshold probabilities, underscoring its superior clinical utility relative to the other models.

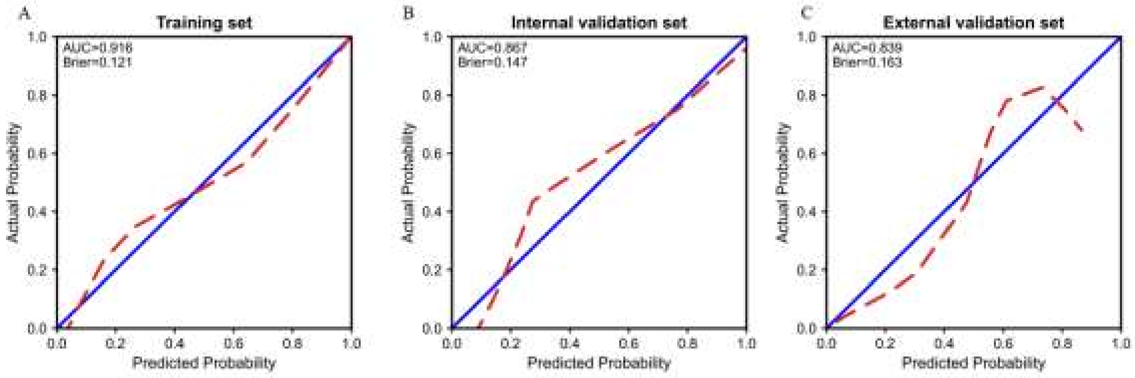

Supplementary Figure S3. Calibration curves of the nomogram in the training (a), internal validation (b) and external validation (c) cohort. The calibration curves indicate that the fusion model is reasonably well calibrated in the training cohort (Brier = 0.121), with minor deviations in internal (Brier = 0.147) and external (Brier = 0.163) validation cohorts, predominantly at extremes of predicted risk. Calibration slopes are close to 1 and intercepts near 0, suggesting that probability estimates are reliable.

The heatmap displays normalized (z-score) radiomic feature profiles of individual patients. Patients are annotated as T790M-negative (red) or T790M-positive (blue). Dendrograms represent clustering of both patients (y-axis) and features (x-axis), revealing distinct radiomic patterns between groups.

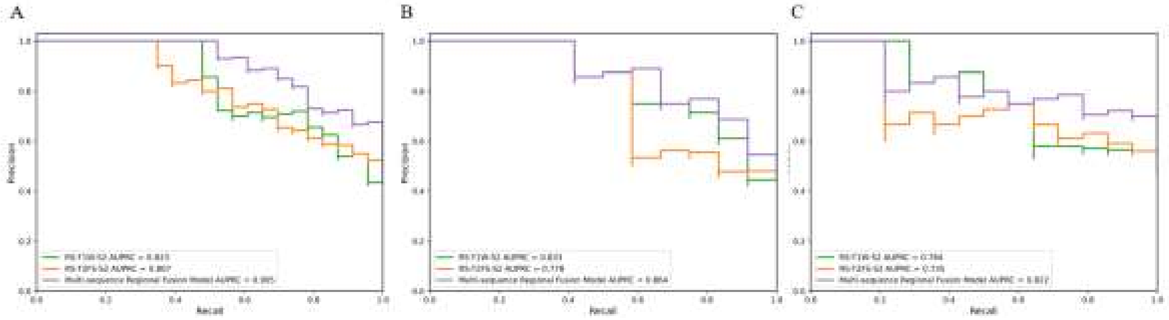

Supplementary Figure S4. The area under the precision–recall curve (AUPRC) of each RS in the training (A), internal validation (B), and external validation (C) sets.

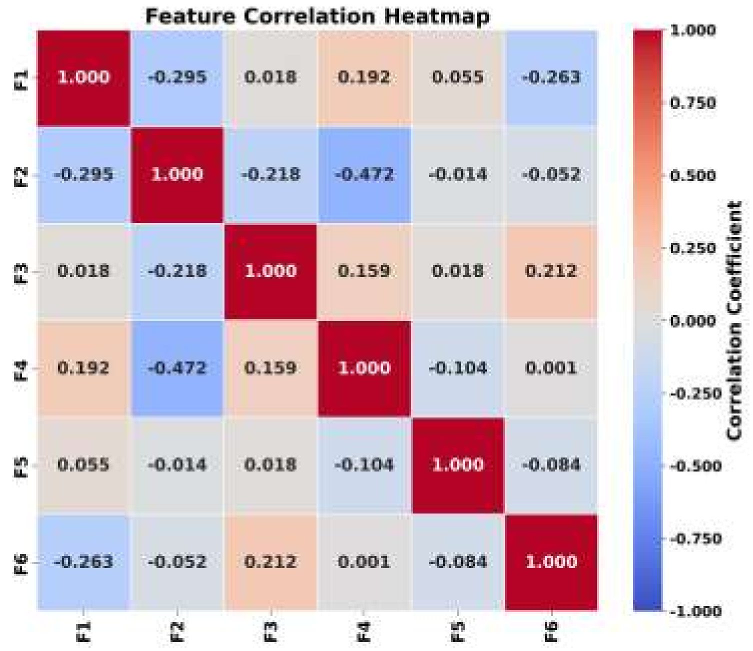

Supplementary Figure S5. Pearson correlation matrix of selected radiomic features. The color scale ranges from blue (strong negative correlation) to red (strong positive correlation), with coefficients from -1 to 1. |r| < 0.3: low correlation; 0.3 ≤ |r| < 0.8: moderate; 0.8 ≤ |r| ≤ 1.0: high correlation.

frontiers

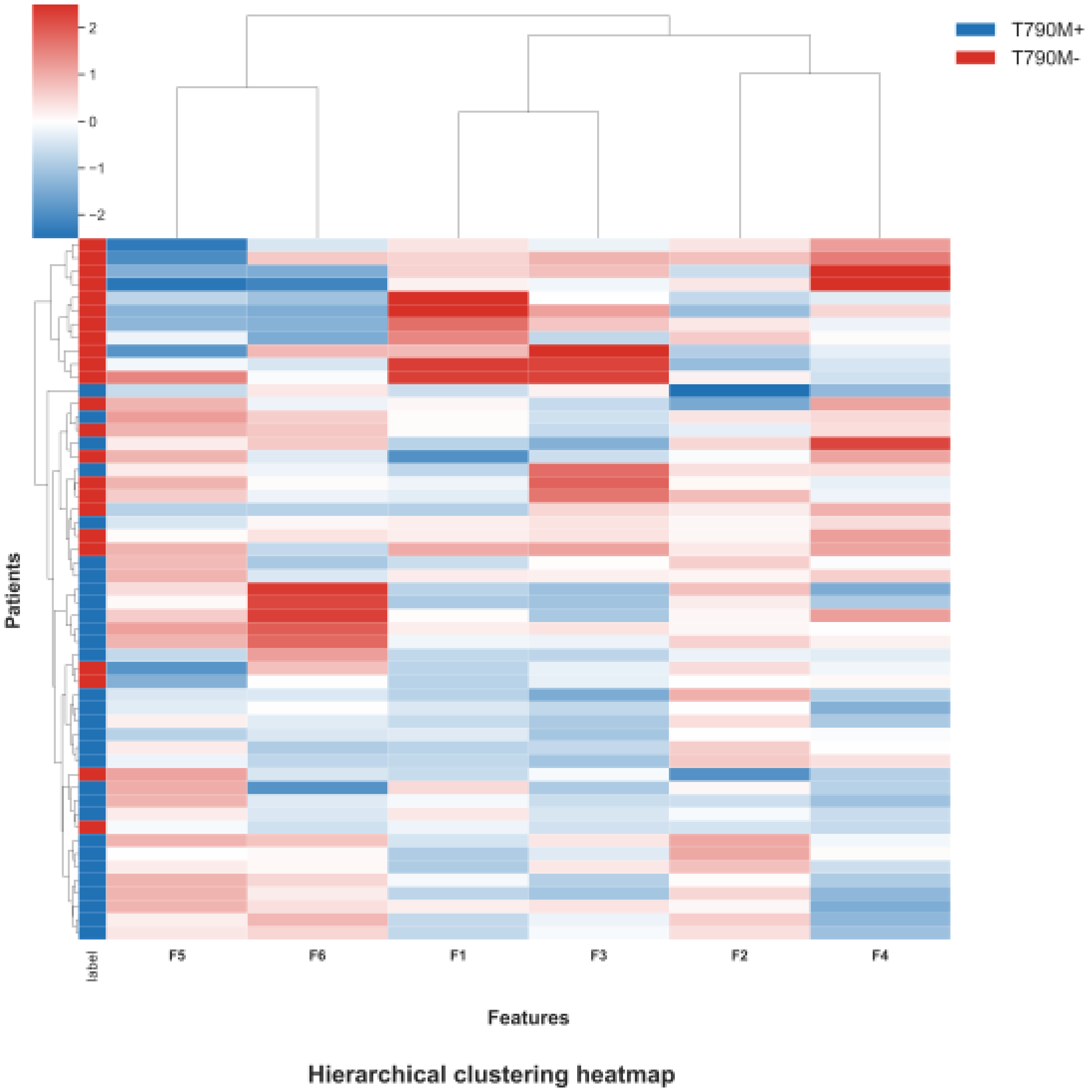

Supplementary Figure S6. Hierarchical clustering heatmap.

Supplementary Method 1. MR Image Preprocessing before Feature Extraction.

Before the feature extraction, preprocessing of the MR images were performed, which include normalization, resampling, discretization and filtering of the images. In our study, the pre-processing steps before feature extraction are shown as:

(***i***)**.** Detailedly, normalized the MRI image by centering it at the mean with standard deviation. Normalization is based on all gray values in the MRI image. The normalizations of the gray level values in the MRI image were performed using the following function: $f(x) = \frac{s(x-\mu_x)}{\sigma_x}$

where:

χ and $f(x)$are the original and normalized intensity, respectively.

μ_x and σ_x are the mean and standard deviation of the image intensity values.

The s is an optional scaling defined by scale and was set to 1 by default.

The parameter "normalize" was set to be true to normalize the image before resampling. The parameter "normalizescale" was set to 100 to determine the scale after normalizing the image.

(***ii***)**.** Afterwards, the parameter "resampledpixelspacing" was set to [1,1,1] to determine the size of the voxel when resampling. And a cubic B-spline interpolator was used to resample MR images.

(***iii***)**.** The parameter "binWidth" was also set to 10 when making a histogram and discretizing the gray level of the images.

(***iv***)**.** Before the feature extraction, the original MR images were transformed with eight types of filters to extract high-dimentional features, which includie wavelet, laplacian of gaussian, square, squareroot, logarithm, exponential, gradient and local binary pattern 2D/3D filters.

(a) **Wavelet filtering:** Decomposes the images into high-frequency components (H) or low-frequency components (L) along three directions. Eight categories of wavelet features were acquired and labeled as HHH, HHL, HLH, LHH, LLL, LLH, LHL, and HLL based on different decomposition orders.
(b) **LoG filtering**: The Laplacian of Gaussian filter (sigma = 1.0, 3.0, 5.0) defines how coarse the emphasized texture should be. Three categories of LoG

features were acquired and labeled as sigma 1.0, sigma 3.0, and sigma 5.0 according to the sigma values.

(c) **Square filtering**: Takes the square of the image intensities and linearly scales them back to the original range.

(d) **SquareRoot filtering:** Takes the square root of the absolute image intensities and scales them back to the original range.

(e) **Logarithm filtering**: Takes the logarithm of the absolute intensity plus 1.

(f) **Exponential filtering**: Takes the exponential, where the filtered intensity is $e^{absolute\ intensity}$.

(g) **Gradient filtering**: Returns the magnitude of the local gradient.

(h) **Local Binary Pattern (LBP) filtering**: Includes 2D and 3D variants (lbp-2D, lbp-3D-k, lbp-3D-m1, lbp-3D-m2). LocalBinaryPattern2D calculates and returns a local binary pattern applied in 2D. LocalBinaryPattern3D calculates and returns local binary pattern maps applied in 3D using spherical harmonics. The last returned image is the corresponding kurtosis map.

Supplementary Table. Settings of parameters in the feature extraction process with the PyRadiomics package.

Parameter	Value
interpolator	3
binWidth	10
normalize	true
normalizeScale	100
sigma	1, 3, 5
resampledpixelspacing	[1,1,1]

Detailed definition and description of the parameters used for the preprocessing of MR images and feature extraction were described in the Pyradiomics documentation (https://pyradiomics.readthedocs.io/en/latest).

Supplementary Formula S1. Multi-sequence Regional Fusion Model for T790M mutation prediction.

l = 27.1643 + 18.5878 × log-sigma-5-0-mm-3D_gldm_DependenceNonUniformityNormalized_T1W - 7.5181 × original_glcm_InverseVariance_T1W + 43.3789 × wavelet-HHH_glcm_MCC_T1W + (25.7972) × exponential_gldm_DependenceNonUniformityNormalized_T2FS - 1.8263 × lbp-3D-m2_firstorder_90Percentile_T2FS - 10.1929 × log-sigma-5-0-mm-3D_glszm_SmallAreaEmphasis_T2FS

Supplementary Table S1. Detailed information on the selected radiomic features

Feature name	Formula	Content
DependenceNon UniformityNormalized (DNN)	$DNN = \frac{\sum_{j=1}^{N_d}(\sum_{i=1}^{N_g} P(i,j))^2}{N_z^2}$	Measures the similarity of dependence throughout the image, with a lower value indicating more homogeneity among dependencies in the image. This is the normalized version of the DLN formula.
InverseVariance	$nverse\ variance = \sum_{k=1}^{N_g-1} \frac{p_{x-y}(k)}{k^2}$	Note that k=0 is skipped, as this would result in a division by 0.
Maximal Correlation Coefficient (MCC)	$MCC = \sqrt{\text{second largest eigenvalue of Q}}$ $Q(i,j) = \sum_{k=0}^{N_g} \frac{p(i,k)p(j,k)}{p_x(i)p_y(k)}$	The Maximal Correlation Coefficient is a measure of complexity of the texture and 0 ≤ MCC ≤ 1. In case of a flat region, each GLCM matrix has shape (1, 1), resulting in just 1 eigenvalue. In this case, an arbitrary value of 1 is returned.
90Percentile	-	The 90th percentile of X.
SmallAreaEmphasis (SAE)	$SAE = \frac{\sum_{i=1}^{N_g}\sum_{j=1}^{N_s}\frac{P(i,j)}{j^2}}{N_z}$	SAE is a measure of the distribution of small size zones, with a greater value indicative of more smaller size zones and more fine textures.

Where:

$p_i \neq 0, \quad p_j \neq 0$

$N.B. \sum_{j=1}^{N_g} s_i$ potentially evaluates to 0 (in case of a completely homogeneous image).

If this is the case, 0 is returned.

μ^4 be the 4th central moment

Note: Detailed image transformation, see supplement method: radiomics features

Supplementary Table S2. The predictive power of the pre-ComBat and post-ComBat radiomics signatures.

	Training			Internal Validation			External Validation		
	**AUC**	**SPE**	**SEN**	**AUC**	**SPE**	**SEN**	**AUC**	**SPE**	**SEN**
Pre-ComBat	0.916 (0.846 - 0.986)	0.867	0.783	0.867 (0.719 - 1.000)	0.800	0.833	0.839 (0.690 – 0.989)	0.812	0.857
Post-ComBat	0.916 (0.846 - 0.986)	0.867	0.783	0.867 (0.719 - 1.000)	0.800	0.833	0.812 (0.623 – 0.972)	0.750	0.857

AUC Area Under the Curve, SPE Specificity, SEN Sensitivity.

Supplementary Table S3. Diagnostic performance of the selected features feature after ComBat harmonization.

Feature	Cohort	Mean ± SD		AUC
		T790M-	T790M+	
log-sigma-5-0-mm-3D_gldm_DependenceNonUniformityNormalized (F1)	Training	0.065 ± 0.014	0.097 ± 0.037	0.772
	Internal validation	0.065 ± 0.014	0.094 ± 0.041	0.739
	External validation	0.073 ± 0.015	0.123 ± 0.062	0.783
original_glcm_InverseVariance (F2)	Training	0.452 ± 0.022	0.420 ± 0.042	0.721
	Internal validation	0.440 ± 0.066	0.420 ± 0.042	0.692
	External validation	0.448 ± 0.031	0.418 ± 0.051	0.689
wavelet-HHH_glcm_ MCC (F3)	Training	0.094 ± 0.018	0.119 ± 0.028	0.778
	Internal validation	0.090 ± 0.217	0.103 ± 0.026	0.775
	External validation	0.131 ± 0.121	0.130 ± 0.038	0.560
exponential_gldm_DependenceNonUniformityNormalized (F4)	Training	0.110 ± 0.044	0.151 ± 0.050	0.732
	Internal validation	0.110 ± 0.032	0.151 ± 0.047	0.731
	External validation	0.109 ± 0.052	0.170 ± 0.139	0.656
lbp-3D-m2_firstorder_90Percentile (F5)	Training	17.167 ± 0.235	16.856 ± 0.503	0.675
	Internal validation	17.107 ± 0.275	16.832 ± 0.500	0.674
	External validation	17.077 ± 0.428	16.936 ± 0.459	0.583
log-sigma-5-0-mm-3D_glszm_SmallAreaEmphasis (F6)	Training	0.262 ± 0.093	0.194 ± 0.073	0.713
	Internal validation	0.259 ± 0.073	0.194 ± 0.123	0.713
	External validation	0.272 ± 0.070	0.221 ± 0.107	0.667

SD standard deviation.

Supplementary Table S4. Diagnostic performance of the selected features before ComBat harmonization.

Feature	Cohort	Mean ± SD		AUC
		T790M-	T790M+	
log-sigma-5-0-mm-3D_gldm_DependenceNonUniformityNormalized (F1)	Training	0.065 ± 0.014	0.097 ± 0.037	0.772
	Internal validation	0.065 ± 0.014	0.094 ± 0.041	0.739
	External validation	0.065 ± 0.014	0.099 ± 0.044	0.759
original_glcm_InverseVariance (F2)	Training	0.452 ± 0.022	0.420 ± 0.042	0.721
	Internal validation	0.440 ± 0.066	0.420 ± 0.042	0.692
	External validation	0.448 ± 0.020	0.427 ± 0.036	0.669
wavelet-HHH_glcm_ MCC (F3)	Training	0.094 ± 0.018	0.119 ± 0.028	0.778
	Internal validation	0.090 ± 0.217	0.103 ± 0.026	0.775
	External validation	0.094 ± 0.017	0.124 ± 0.030	0.817
exponential_gldm_DependenceNonUniformityNormalized (F4)	Training	0.110 ± 0.044	0.151 ± 0.050	0.732
	Internal validation	0.110 ± 0.032	0.151 ± 0.047	0.731
	External validation	0.111 ± 0.046	0.155 ± 0.053	0.740
lbp-3D-m2_firstorder_90Percentile (F5)	Training	16.856 ± 0.503	17.167 ± 0.235	0.675
	Internal validation	17.167 ± 0.235	16.856 ± 0.503	0.674
	External validation	17.176 ± 0.224	16.853 ± 0.509	0.680
log-sigma-5-0-mm-3D_glszm_SmallAreaEmphasis (F6)	Training	0.262 ± 0.093	0.194 ± 0.073	0.713
	Internal validation	0.259 ± 0.073	0.194 ± 0.123	0.713
	External validation	0.258 ± 0.095	0.200 ± 0.082	0.649

SD standard deviation.

Supplementary Table S5. Comprehensive Model Performance Analysis: AUPRC, PPV, NPV, and F1 Scores Across Multiple Phases

	Training				Internal validation				External validation			
	**AUPRC (95%CI)**	**PPV**	**NPV**	**F1**	**AUPRC (95%CI)**	**PPV**	**NPV**	**F1**	**AUPRC (95%CI)**	**PPV**	**NPV**	**F1**
RS - T1W - S2	0.823 (0.710–0.923)	0.720	0.720	0.750	0.833 (0.674 - 0.966)	0.714	0.846	0.769	0.784 (0.581 – 0.899)	0.688	0.786	0.733
RS - T2FS - S2	0.807 (0.693 - 0.912)	0.727	0.774	0.711	0.778 (0.612 - 0.928)	0.875	0.737	0.700	0.735 (0.562 – 0.903)	0.750	0.722	0.692
Multi-sequence Regional Fusion Model	0.905 (0.824 - 0.971)	0.818	0.839	0.800	0.864 (0.724 - 0.980)	0.769	0.857	0.800	0.822 (0.596 – 0.975)	0.800	0.867	0.828

S1 subregion 1, S2 subregion 2, AUPRC Area Under the Precision-Recall Curve, PPV Positive Predictive Value, NPV Negative Predictive Value, F1 F1 Score (or F1-Score, a harmonic mean of Precision and Recall).
